# Single‐Pulse Femtosecond Laser 3D Multi‐Foci Light‐Field Engineering via Multi‐Layer Noise Threshold Filtering

**DOI:** 10.1002/advs.77988

**Published:** 2026-09-28

**Authors:** Shouyu Wu, Ruiyang Wang, Lan Jiang, Mengyao Tian, Yongjiu Yuan, Junrui Wu, Liang Zhao, Ruochen Zhang, Ziqian Ning, Xin Li

**Affiliations:** ^1^ Laser Micro/Nano‐Fabrication Laboratory School of Mechanical Engineering Beijing Institute of Technology Beijing P. R. China; ^2^ Complex and Microscopic Structure Fabrication Technologies National‐level Innovation Center Beijing P. R. China; ^3^ Yangtze Delta Region Academy of Beijing Institute of Technology Jiaxing P. R. China; ^4^ Beijing Institute of Technology Chongqing Innovation Center Chongqing P. R. China

**Keywords:** femtosecond laser, micro/nano manufacturing, multi‐layer noise threshold filtering, multi‐layer over‐sampling algorithm, three‐dimensional light‐field shaping

## Abstract

Three‐dimensional processing is a key trend in micro/nano manufacturing, with widespread applications in photonics, manufacturing, and optical storage. However, simultaneously achieving high fabrication resolution, throughput, and quality for three‐dimensional structures remains challenging. Here, we propose a three‐dimensional multi‐foci light‐field shaping and high‐throughput micro/nano manufacturing method based on a single pulse. This method combines a multi‐layer noise threshold filtering algorithm with a multi‐layer over‐sampling algorithm to generate a three‐dimensional multi‐foci light field with up to 5,400 laser foci across six layers using a dual‐spatial‐light‐modulator parallel system. In processing experiments, a representative single‐spatial‐light‐modulator light field contains 1,200 laser foci per pulse. Under the dual‐spatial‐light‐modulator parallel system, this configuration corresponds to 2,400 processing points per incident pulse, enabling a demonstrated processing throughput of 24 000 000 processing points s^−1^. In addition, the method achieves an isolated smallest feature of approximately 50 nm and demonstrates high manufacturing flexibility and broad material compatibility across inorganic, organic, and hydrogel materials. This strategy provides a precise, scalable, and efficient route for high‐quality three‐dimensional micro/nano manufacturing.

## Graphical abstract

This study presents a single‐pulse femtosecond laser strategy for three‐dimensional multi‐foci light‐field shaping and high‐throughput micro/nano manufacturing. The multi‐layer noise threshold filtering algorithm improves focal quality, while the multi‐layer over‐sampling algorithm increases focal density. This strategy provides a precise, scalable, and efficient route for high‐quality three‐dimensional micro/nano manufacturing

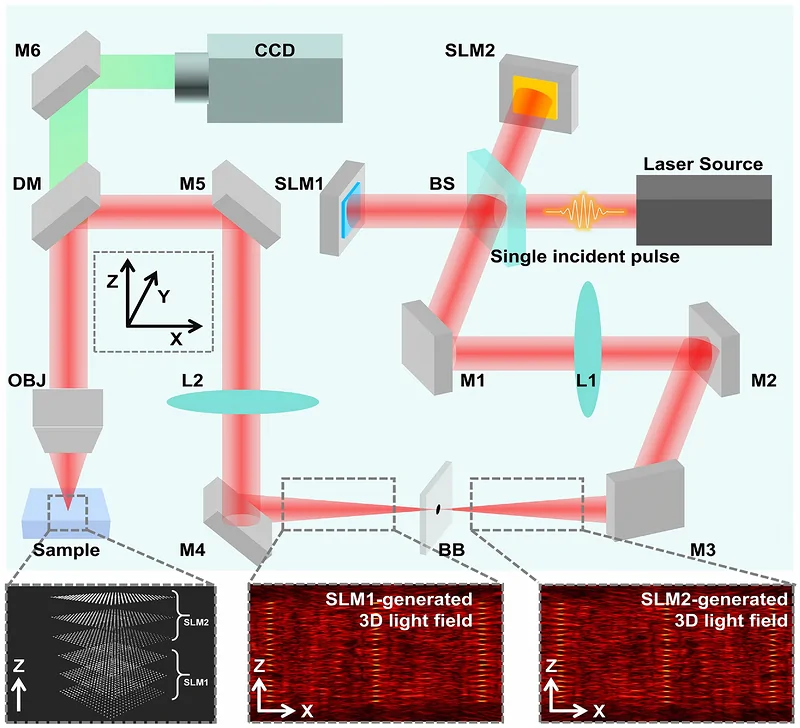

## 1 Introduction

Precise and efficient manufacturing of 3D structures for driving the miniaturization and integration of devices is increasingly vital [1, 2, 3, 4]. However, this trend poses significant challenges to manufacturing technologies, requiring methods capable of producing 3D micro/nanostructures with both high fabrication resolution and high processing throughput, which existing techniques struggle to achieve simultaneously [5, 6]. Although traditional photolithography offers excellent fabrication resolution and processing throughput, its mask dependence, process complexity, and poor manufacturing flexibility indirectly compromise its manufacturing efficiency [7]. Electron beam lithography [8] and ion beam lithography [9] offer extremely high fabrication resolution, but their processing throughput is generally low, making it difficult to meet the demands of high integration. Laser spatial shaping enables the transformation of a single laser focus into a multi‐focus light field, which can significantly increase the processing throughput while maintaining resolution [10]. It is widely applied in fields such as energy storage [11, 12], manufacturing [13, 14], and optical storage [15]. However, current work on single‐pulse spatial shaping has largely remained confined to 2D manipulation. The generation of 3D light fields suitable for micro/nano manufacturing remains a significant challenge.

The primary devices currently capable of achieving laser spatial shaping include the diffractive optical element (DOE), the digital micromirror device (DMD), and the spatial light modulator (SLM) [16]. Among these, the DOE functions similarly to fixed laser masks and faces the same limitations as traditional photolithography techniques [17]. The DMD can achieve relatively high fabrication resolution and processing throughput [18, 19]. However, due to its amplitude modulation mechanism, its energy utilization efficiency is relatively low, and it is limited to single‐pulse 2D shaping. The SLM modulates the phase of the laser in combination with algorithms such as Fourier transform, Fresnel diffraction, or angular spectrum methods [20, 21, 22]. It has been demonstrated to possess excellent spatial shaping and processing capabilities, particularly the potential for single‐pulse 3D light‐field shaping [23, 24, 25, 26]. The natural propagation of the laser along the axis results in a considerable amount of energy inter‐plane crosstalk. This results in a reduction in the quality of the inter‐plane light field and affects the uniformity of the processed structures [27]. In general, an increased number of layers and reduced inter‐layer spacing exacerbate crosstalk. Staggering patterns across layers can partially mitigate crosstalk effects, but at the expense of significantly compromising 3D spatial capacity [28]. Consequently, resolving the adverse impact of high inter‐plane crosstalk within three‐dimensional light fields is crucial for enabling true multi‐layer, single‐pulse laser manufacturing.

We present a single‐pulse three‐dimensional multi‐foci light‐field shaping and high‐throughput micro/nano manufacturing method to address these challenges. The method combines a multi‐layer noise threshold filtering algorithm (M‐NTF), which suppresses inter‐plane crosstalk, with a multi‐layer over‐sampling algorithm (M‐OS), which reduces the calculated focal‐spot size and spacing. In numerical light‐field shaping, the dual‐SLM parallel system enables up to 20 layers or, alternatively, up to 5,400 laser foci across six layers within two split sub‐pulses derived from one incident pulse. In laser processing, a representative 3 × 20 × 20 light field was experimentally demonstrated using one SLM, corresponding to 1,200 laser foci per pulse. With the dual‐SLM parallel system, this configuration yields 2,400 processing points per pulse and a demonstrated processing throughput of 24 000 000 processing points per second. Under 9× over‐sampling, repeated measurements yielded a mean fabricated feature size of approximately 94 nm, with an isolated smallest feature of approximately 50 nm also observed. The method further demonstrated high manufacturing flexibility and broad material compatibility.

## 2 Results and Discussion

### 2.1 The Method Schematic of 3D Multi‐Foci Light‐Field Shaping and High‐Throughput Micro/Nano Manufacturing

The method presented in this study can effectively transform a single‐pulse Gaussian laser into a 3D light field, as illustrated in Figure 1a. This is achieved through our proposed multi‐layer noise threshold filtering algorithm (M‐NTF), which effectively suppresses the energy crosstalk between the layers. This ensures that the energy density in non‐processing regions remains significantly lower than that in processing regions, thereby guaranteeing processing quality. On the other hand, combined with our proposed multi‐layer over‐sampling algorithm (M‐OS), it further enables precise control over calculated focal‐spot size and spacing. This enhances the accuracy and quantity of laser foci in the 3D light field, thereby guaranteeing fabrication resolution and processing throughput. The three‐dimensional reconstruction of the processing result obtained using the 3D multi‐foci light field is shown in Figure 1b, and the corresponding details are provided in Figure S1.

**FIGURE 1:**
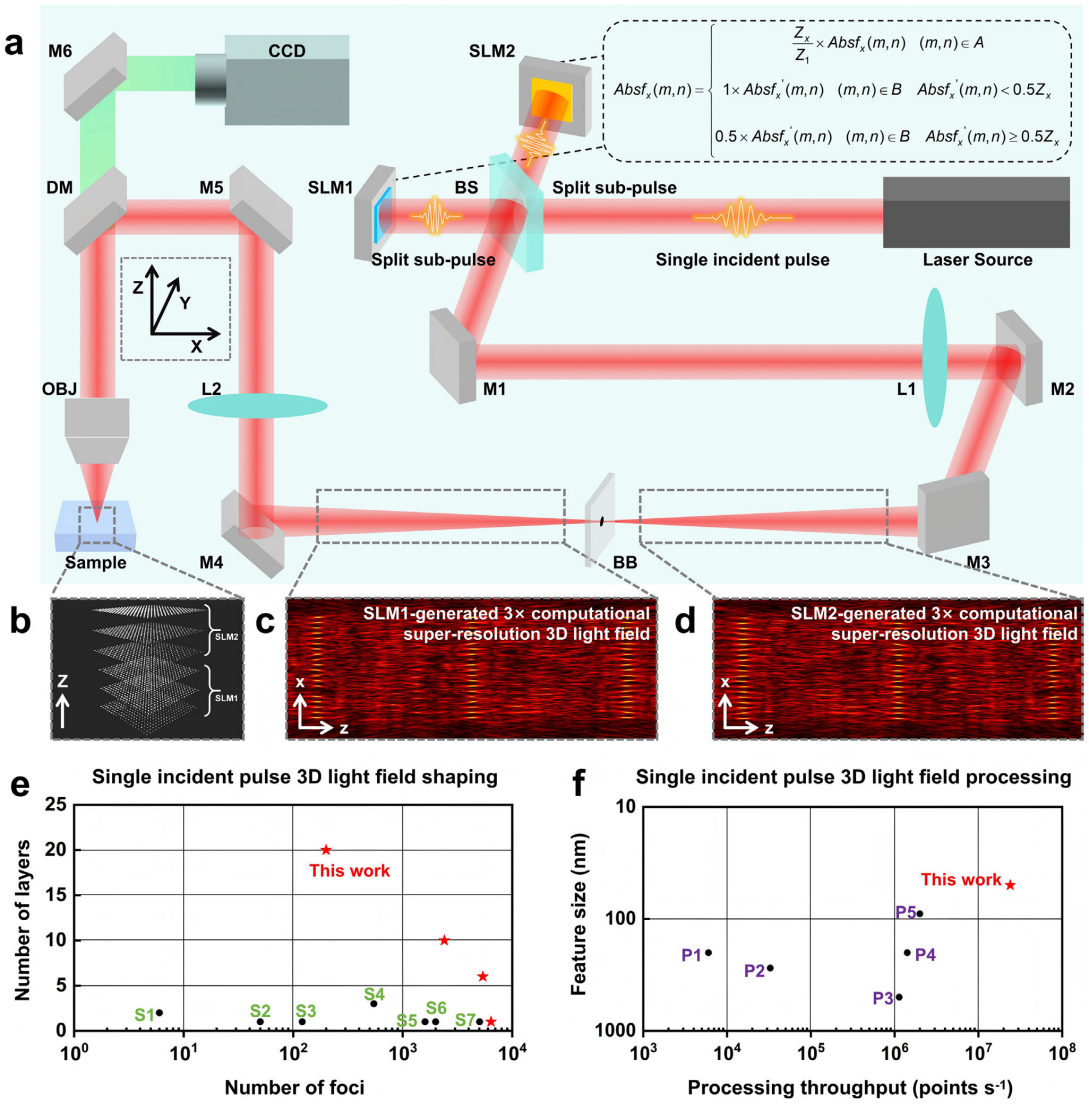
Schematic of 3D multi‐foci light‐field shaping and high‐throughput micro/nano manufacturing by two split sub‐pulses derived from one incident Gaussian laser pulse. (a) Optical setup diagram of 3D multi‐foci light‐field shaping and high‐throughput micro/nano manufacturing with the dual‐SLM parallel system. BS, beam splitter; SLM, spatial light modulator; M, mirror; L, plano‐convex lens; BB, beam block; DM, dichroic mirror; OBJ, objective lens. (b) Three‐dimensional reconstruction of the 2,400 processing points fabricated in PET by the 3D multi‐foci light field. (c) Three‐dimensional light field distribution generated by SLM1 and its position in the optical setup. (d) Three‐dimensional light field distribution generated by SLM2 and its position in the optical setup. (e) Comparison of the number of layers and the number of foci by single‐pulse 3D multi‐foci light‐field shaping. (f) Comparison of the smallest individually observed feature size and processing throughput in single‐pulse 3D light‐field micro/nano manufacturing. Literature references are as follows: S1, Li et al. [25]; S2, Geng et al. [30]; S3, Arnoux et al. [31]; S4, Ren et al. [27]; S5, Yang et al. [17]; S6, Ouyang et al. [10]; S7, Su et al. [32]; P1, Li et al. [25]; P2, Obata et al. [33]; P3, Arnoux et al. [31]; P4, Geng et al. [30]; P5, Ouyang et al. [10]. S and P denote references used for spatial‐capacity and processing‐performance comparisons, respectively.

Specifically, the laser source emits a single‐pulse femtosecond laser beam, which, in the dual‐SLM configuration, is divided by the beam splitter (BS) into two split sub‐pulses with an energy ratio of 1:1. The two sub‐pulses are independently directed to SLM1 (spatial light modulator 1) and SLM2 (spatial light modulator 2) for phase modulation and reflection, and are then recombined by the BS. The optical path length from the BS to SLM1 is 1 mm shorter than that from the BS to SLM2; therefore, the total round‐trip optical path of the SLM1 arm is 2 mm shorter than that of the SLM2 arm. This introduces a temporal delay of approximately 6.7 ps between the two sub‐pulses. Since the pulse duration of each sub‐pulse is 250 fs, the two sub‐pulses do not temporally overlap in the optical path and therefore do not produce coherent interference. Instead, they function as two temporally separated but parallel split sub‐pulses. The detailed alignment and operation of the dual‐SLM parallel system are described in Note S2 [29].

A plano‐convex lens (L1) with a focal length of 750 mm is placed 750 mm after SLM1. After another propagation distance of 750 mm, a beam block (BB) with a 1 mm‐diameter black spot at its center is placed to simultaneously block the zero‐order light generated by both SLM1 and SLM2. A three‐layer planar light field modulated by a negative‐lens phase is loaded onto SLM1, so that the corresponding three‐layer planar light field is generated after the zero‐order light position, as shown in Figure 1c. Correspondingly, a three‐layer planar light field modulated by a positive‐lens phase is loaded onto SLM2, so that the corresponding three‐layer planar light field is generated before the zero‐order light position, as shown in Figure 1d. This design enables the lower three layers to be processed before the upper three layers in the parallel processing process, thereby preventing the structures generated by the earlier‐arriving pulse from degrading the processing quality of the later‐arriving pulse. A second plano‐convex lens (L2) with a focal length of 750 mm is placed 750 mm after the BB. L1 and L2 together form a 4f system, which relays the laser fields from SLM1 and SLM2 to the position before the focusing objective. An objective lens (Olympus, 20×, NA = 0.45) is placed 750 mm after L2 to focus the laser into the interior of the material for micro/nano processing.

Here, we define the SLM plane as the incident light‐field region, the BB plane as the pre‐generated light‐field region, and the sample plane as the processing region. In the incident light‐field region, a 1080 × 1080‐pixel phase hologram is loaded at the center of the SLM, whose full device resolution is 1920 × 1080 pixels with a pixel pitch (the intrinsic pixel pitch of the SLM device, measured in units of length) of 8 µm. In the pre‐generated light‐field region, this configuration corresponds to a generated light field of 1080 × 1080 pixels with a pixel spacing (the spacing between adjacent sampling pixels of the light field, measured in units of length) of 89.4 µm in the absence of over‐sampling. Because M1–M5, L1, and L2 in the optical path all have a diameter of 2 inches (5.08 cm), only a circular beam region with a diameter corresponding to approximately 568 (5.08 cm/89.4 µm) pixels can pass through the system. This circular region can be cropped into a maximum inscribed square region of approximately 401 × 401 pixels. To further ensure processing stability, we only use the central 300 × 300‐pixel region as the defined region for pattern design. Since distortion already appears near the edge of the 300 × 300‐pixel region, simply increasing the lens diameter to allow a larger region to pass through the system would have limited practical value before the distortion issue is addressed. In the processing region, under the above optical configuration, the central 300 × 300‐pixel cropped region in the pre‐generated light‐field plane is scaled to a light field with a pixel spacing of 800 nm. Finally, the maximum number of shaped layers and foci achieved in the pre‐generated light‐field region is shown in Figure 1e, while the maximum processing throughput and the smallest individually observed fabricated feature size (the major‐axis length of the fitted ellipse obtained by fitting the boundary of each fabricated structure) achieved in the processing region are shown in Figure 1f. Compared with the shaping capacities (S1–S7) and processing performances (P1–P5) reported in previous studies, our method exhibits significant advantages in both aspects. In addition, a computational benchmark of the proposed M‐NTF + M‐OS framework against direct phase superposition (DPS) and mixed‐region amplitude freedom (MRAF) is provided in Figure S2 and Table S1.

### 2.2 The 3D Light Field Generation by Multi‐Layer Noise Threshold Filtering Algorithm

The traditional Gerchberg–Saxton (GS) algorithm [34] (Note S4) for laser spatial shaping operates through iterative 2D Fourier transforms, cycling between the incident light field and pre‐generated light field to achieve pre‐generated patterning. However, the basic GS algorithm is limited to establishing a connection between a single incident light field layer and a single pre‐generated light field layer (Figure 2a), and therefore cannot generate a 3D light field. By loading Fresnel phase onto the incident light field, the position of the pre‐generated light field layer can be adjusted to achieve multi‐layer light‐field shaping (Figure 2b). However, this results in severe crosstalk between light field layers. Therefore, the M‐NTF is proposed.

**FIGURE 2:**
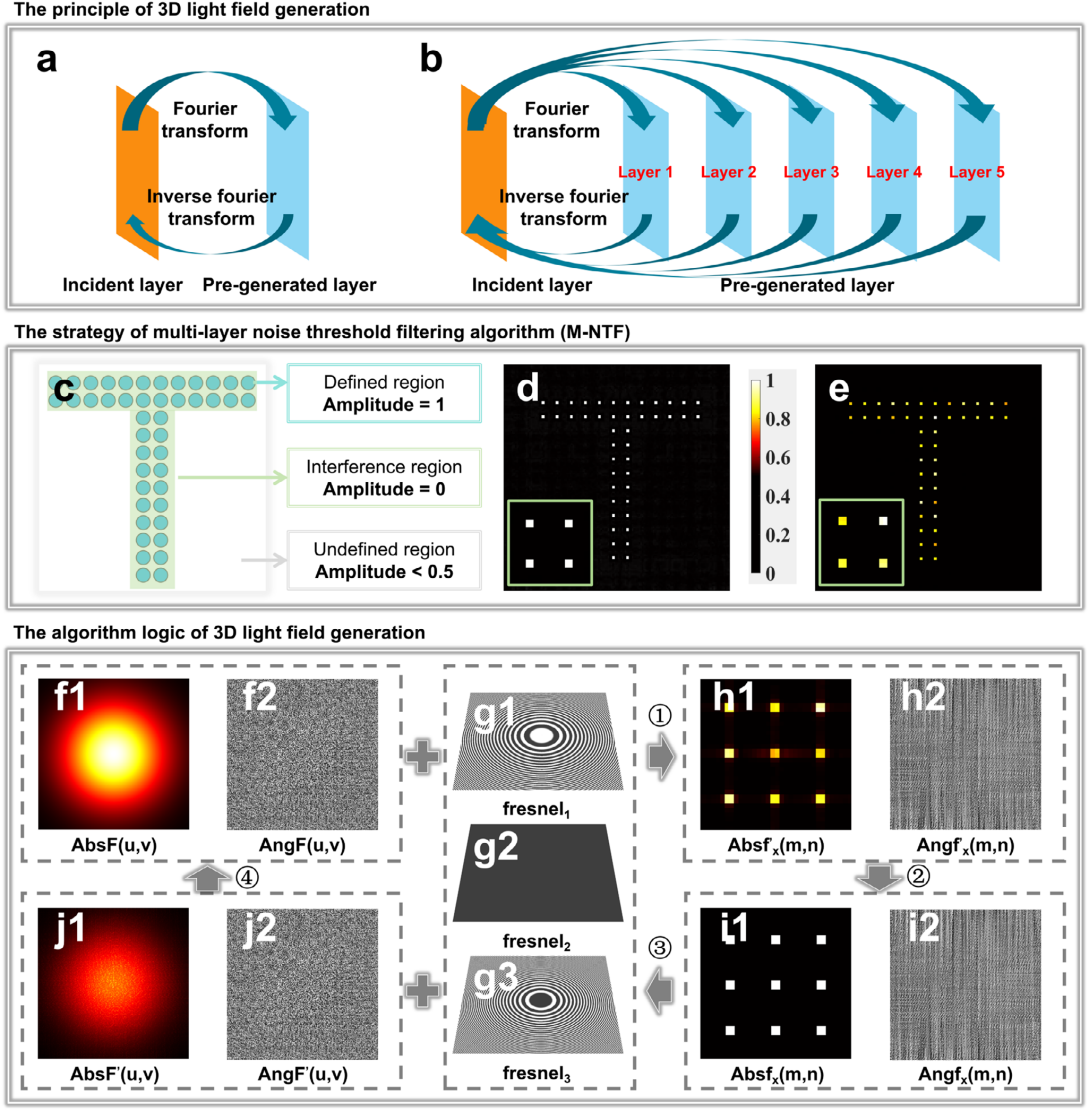
Principle and algorithm logic of 3D light‐field generation. (a,b) Principles of 2D and 3D light‐field generation, respectively. (c) Division of each pre‐generated light‐field layer into the defined region, interference region, and undefined region, shown in blue, green, and white, respectively. (d,e) Simulated light‐field distributions with and without M‐NTF, respectively. (f1,f2) Amplitude of the actual incident light field and phase loaded onto the SLM. (g1–g3) Fresnel phases corresponding to the first, second, and third light‐field layers. (h1,h2) Amplitude and phase of the calculated pre‐generated light field. (i1,i2) Amplitude and phase of the actual pre‐generated light field after amplitude replacement. (j1,j2) Amplitude and phase of the calculated incident light field.

The fundamental concept underpinning this algorithm involves the division of each pre‐generated light field layer into three distinct regions: a defined region, an interference region, and an undefined region. Each of these regions is subject to a unique convergence strategy (Figure 2c). In the defined region, corresponding to the target processing pattern, the relative amplitude is strictly constrained to 1 to ensure structural consistency and fabrication quality. In the interference region, which is adjacent to the defined region, the relative amplitude is strictly constrained to 0, forming a buffer that prevents neighboring in‐plane crosstalk from disturbing the defined region. Notably, the interference region is manually specified by the user according to a fixed geometric rule, rather than derived from the diffraction characteristics of the target light field. In the undefined region, the relative amplitude is constrained below 0.5, corresponding to a relative energy density below 0.25. This lower‐priority constraint allows residual background noise to remain, provided that it stays below the processing threshold of transparent materials and therefore does not contribute to fabrication [35]. In the current implementation, the main design parameters are determined according to the target 3D light‐field pattern and optical configuration, whereas some optimization parameters, including iteration number, convergence weights, and relative amplitude in the undefined region, are manually adjusted based on the target light‐field design and experimental feedback.

The light field simulation result achieved using M‐NTF is demonstrated in Figure 2d. In comparison with the light field simulation that does not incorporate M‐NTF (Figure 2e), although there is some crosstalk in the undefined region, the energy density within the defined region is uniformly consistent. Because the residual intensity in the undefined region remains below the material processing threshold, it does not deteriorate the fabricated structures. Therefore, M‐NTF improves the effective processing quality by prioritizing the defined and interference regions. The details of the M‐NTF logic are as follows. The comprehensive lists of all nomenclature and equations are provided in Note S5 and Table S2.

First, based on the amplitude of the actual incident light field and the phase loaded onto the SLM (Figure 2f), the Fresnel phase (Figure 2g) is added, and a forward Fourier transform is applied to obtain the distribution of the calculated pre‐generated light field (Figure 2h). Second, the amplitude of the calculated pre‐generated light field is replaced with that of the actual pre‐generated light field, while its phase is retained (Figure 2i). Third, an inverse Fourier transform is applied to obtain the distribution of the calculated incident light field (Figure 2j). The Fresnel phase is then subtracted from the phase, and the amplitude of the calculated incident light field is replaced with that of the actual incident light field. Fourth, steps 1–3 are repeated using different Fresnel phases. Finally, steps 1–4 are iterated until the amplitude of the calculated pre‐generated light field matches that of the actual pre‐generated light field for each layer. Further details of the algorithm are provided in Note S6. A quantitative evaluation of the in‐plane intensity uniformity and inter‐plane crosstalk suppression achieved by M‐NTF is provided in Figures S3–S5.

### 2.3 The 3D Light Field Extension by Multi‐Layer Over‐Sampling Algorithm

With the quality of the 3D light field assured, our focus shifts to enhancing its fabrication resolution and processing throughput. First and foremost, the primary challenge lies in reducing the calculated focal‐spot size and spacing to enable a single‐pulse 3D light field to accommodate more densely distributed and smaller laser foci. Therefore, the M‐OS is proposed. The fundamental concept underpinning this algorithm involves the spatial Fourier transform. Over‐sampling the incident light field (extend the pixel space (the pixel‐domain extent of the light field, measured by the number of pixels)) enables computational super‐resolution (not optical super‐resolution) of the pre‐generated light field (reduce the pixel spacing) [36]. The details of the M‐OS logic are as follows.

For the incident layer (Figure 3a,b), the incident laser is loaded onto the SLM. Based on our SLM model, it is resolved into a discrete incident light field of 1,080 × 1,080 pixels, with a pixel pitch of 8 × 8 µm^2^. A discrete Fourier transform of the discrete incident light field is performed to yield the discrete pre‐generated light field (Figure 3c,d). Under a 750 mm focal length plano‐convex lens, the discrete pre‐generated light field is 1,080 × 1,080 pixels, with an effective pixel size of 89.4 × 89.4 µm^2^. Each laser focus occupies a 2 × 2 pixel space. The incident layer is then oversampled to 3,240 × 3,240 pixels while maintaining the same pixel size. In this case, the light field distribution of the central 1,080 × 1,080 pixels remains unchanged, while the amplitude in the peripheral part is set to zero (Figure 3e,f). A discrete Fourier transform of the over‐sampling discrete incident light field is performed to yield the computational super‐resolution discrete pre‐generated light field (Figure 3g,h). The computational super‐resolution discrete pre‐generated light field is 3,240 × 3,240 pixels, with an effective pixel size of 29.8 × 29.8 µm^2^. Each laser focus in the light field therefore occupies a 6 × 6 pixel space. The detailed calculation formula is included in Note S8. At this point, the 3× computational super‐resolution of the pre‐generated light field can be realized. In principle, higher over‐sampling factors can further refine the numerical representation of the pre‐generated light field, while the practically achievable processing precision is ultimately limited by the optical aperture, phase aberrations, pulse energy, and material response. Comparison of the same laser light field distribution at different over‐sampling factors is shown in Figure S6.

**FIGURE 3:**
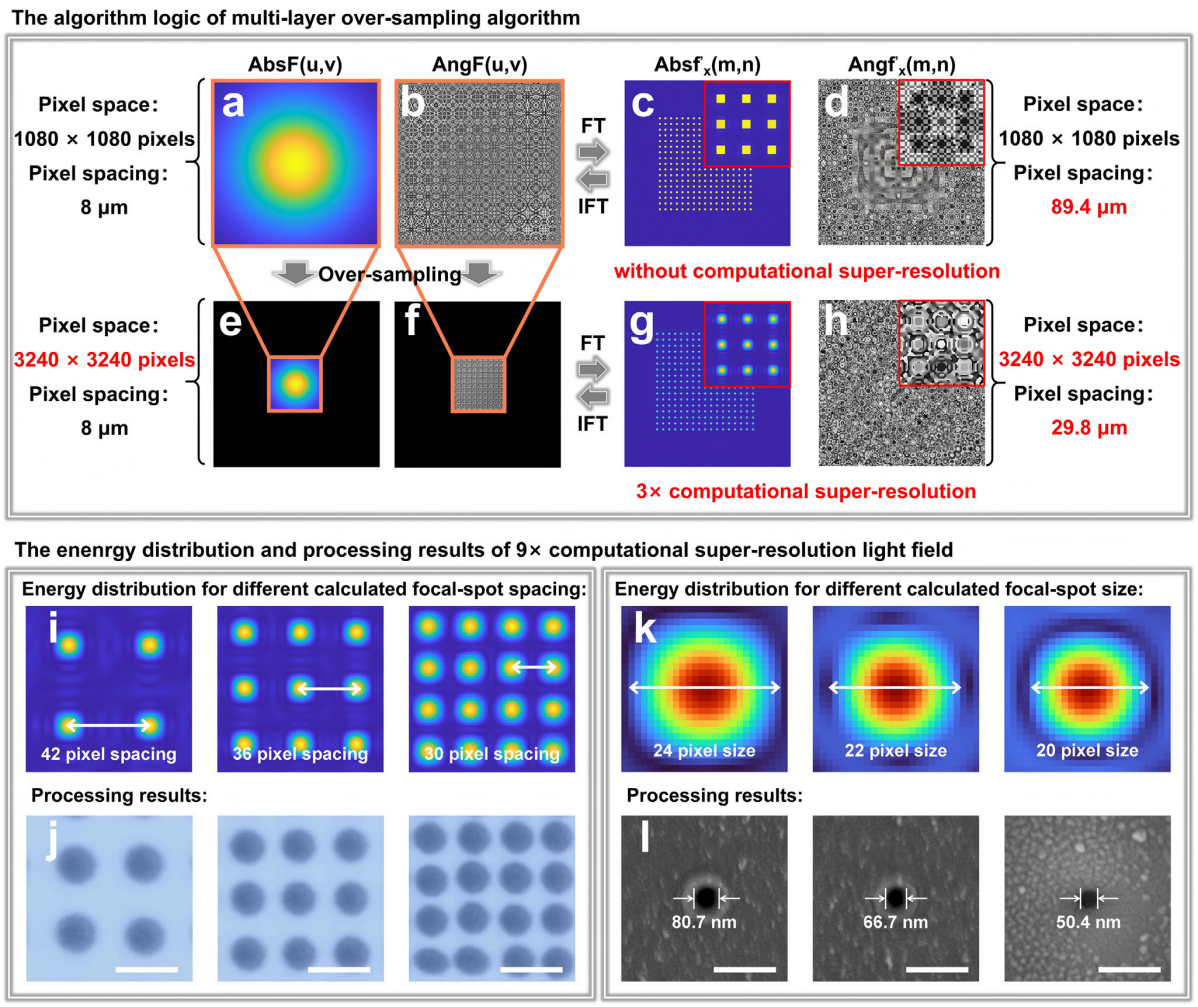
Principle and algorithm logic of 3D light‐field extension. (a,b) Amplitude and phase distributions of the incident computational conventional‐resolution light field. (c,d) Amplitude and phase distributions of the pre‐generated computational conventional‐resolution light field. (e,f) Amplitude and phase distributions of the incident 3× computational super‐resolution light field. (g,h) Amplitude and phase distributions of the pre‐generated 3× computational super‐resolution light field. (i,j) Amplitude distribution and corresponding processing results of 9× computational super‐resolution light fields with different calculated focal‐spot spacing. (k,l) Amplitude distribution and corresponding processing results of 9× computational super‐resolution light fields with different calculated focal‐spot sizes. All light fields in Figure 3 correspond to SLM1. The processing results in (j) were obtained using an Olympus 20× objective lens (NA = 0.45), and those in (l) were obtained using an Olympus 100× objective lens (NA = 0.95). Scale bars, 4 µm (j) and 200 nm (l).

The simulated 9× computational super‐resolution light‐field distributions with different pixel spacings are shown in Figure 3i. Meanwhile, the actual laser processing results are shown in Figure 3j. It is worth noting that the calculated focal‐spot spacing can be optimized to as low as 30 pixels, which is equivalent to a calculated focal‐spot spacing of 3.33 pixels in the computational conventional‐resolution light field. It is evident that this objective cannot be achieved in the absence of computational super‐resolution calculations, given that the minimum pixel spacing that can be attained using computational conventional‐resolution optimization is 4 pixels (Figure S7). Moreover, the calculated focal‐spot size can be further compressed to 20 pixels under 9× computational super‐resolution (Figure 3k). An isolated smallest fabricated feature of approximately 50 nm is shown in Figure 3l. In the repeated measurements under the 9× over‐sampling condition, the mean fabricated feature size was 94.06 ± 24.02 nm, with a minimum value of 58.75 nm within the repeated‐measurement dataset. Details of calculated light fields, measured light fields, and processing results are shown in Figures S8 and S9. It should be noted that the measured light fields presented were characterized before the focusing objective and therefore represent the pre‐objective multi‐foci intensity distributions rather than direct measurements of the final high‐NA focal point‐spread function at the sample.

### 2.4 The Processing Capability for the Single‐Pulse 3D Multi‐Foci Light Field

After specifying the minimum calculated focal‐spot size and spacing, we further investigated the maximum spatial capacity of three‐dimensional light‐field shaping and processing. For the spatial capacity of 3D multi‐foci light‐field shaping, the simulation results of discrete dot arrays generated using SLM1 are shown below, and the corresponding detailed light‐field simulations are provided in Figure S10.

A single layer achieves 1 × 40 × 40 laser foci with a calculated focal‐spot spacing of 10 pixels (3× computational super‐resolution). Three layers achieve 3 × 30 × 30 laser foci with a calculated focal‐spot spacing of 30 pixels (3× computational super‐resolution). Five layers achieve 5 × 10 × 10 laser foci with calculated focal‐spot spacing of 90 pixels (3× computational super‐resolution). Ten layers achieve 10 × 10 × 1 laser foci with calculated focal‐spot spacing of 90 pixels (3× computational super‐resolution).

The greater the number of 3D light field layers, the fewer laser foci each layer can accommodate. Otherwise, the processing quality will deteriorate. Considering the number of processing foci, the degree of edge distortion, and the optical energy utilization efficiency, we finally selected the 3 × 20 × 20 discrete 3D light field shown in Figure 4a as the processing light field for demonstrating the maximum throughput. It consists of three layers (Figure 4b), each containing 20 × 20 laser foci, with a calculated focal‐spot spacing of 30 pixels (Figure 4c). The processing results of each layer are shown in Figure 4d,e. Details of calculated light fields, measured light fields, and processing results are shown in Figures S11–S13. In practical single‐pulse processing, optical distortion caused by the refractive‐index mismatch between air and the workpiece material must be considered. This distortion becomes more pronounced as the focal depth inside the material increases. Therefore, according to the specific workpiece material and processing depth, we superimpose an aberration‐correction phase onto the corresponding 3D light‐field phase pattern [37]. The detailed implementation and results are provided in Table S3 and Figure S14.

**FIGURE 4:**
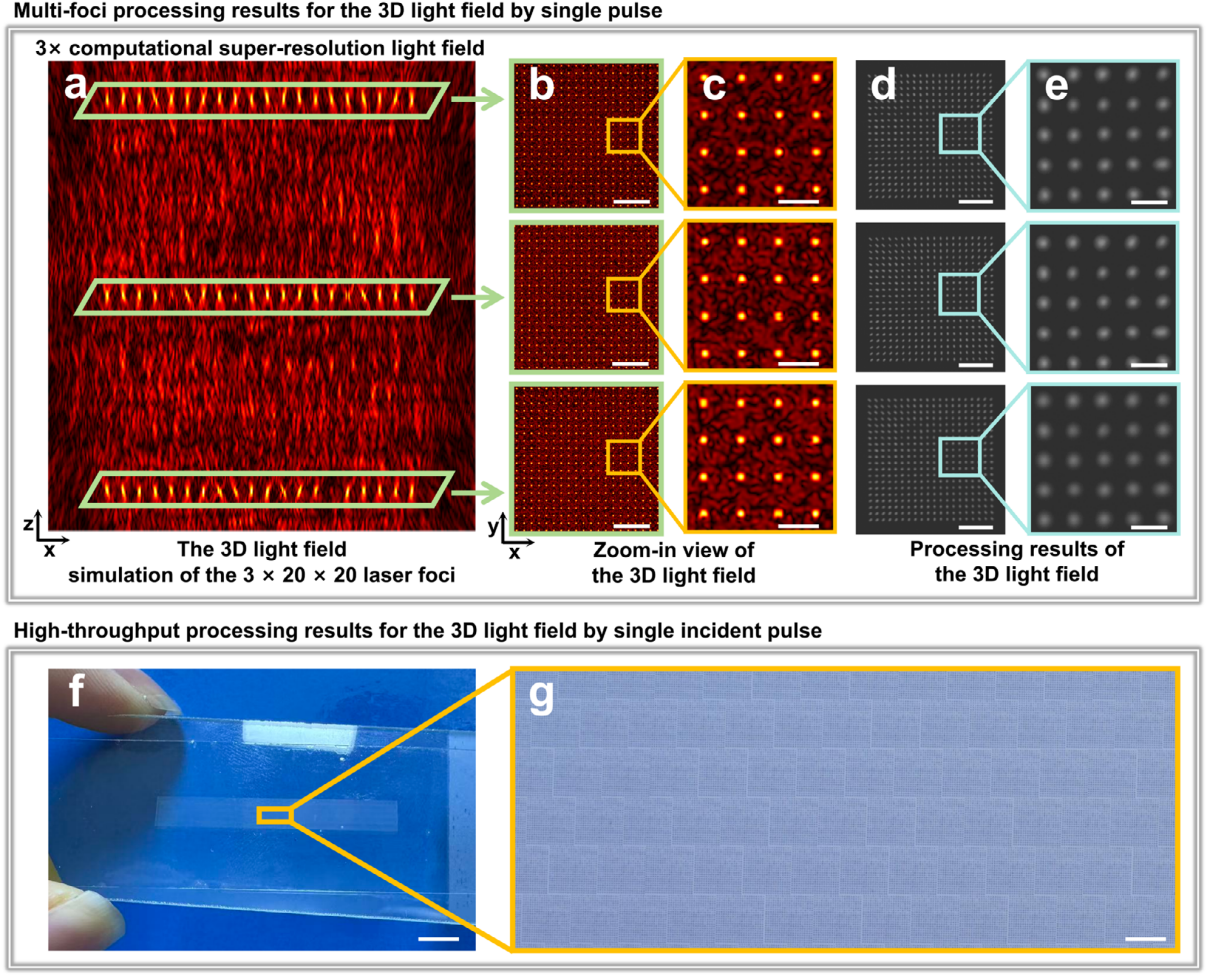
Processing results of the single‐pulse 3D light field. (a) Energy distribution of the discrete 3D light field. (b) Zoom‐in view of (a). (c) Zoom‐in view of (b). (d) Processing results of the discrete 3D light field. (e) Zoom‐in view of (d). (f) Macro view of the processing area. (g) Zoom‐in view of (f). The results in (a–e) correspond to SLM1 with 3× computational super‐resolution and were processed using an Olympus 20× objective lens (NA = 0.45). The results in (f,g) correspond to the dual‐SLM parallel system with 3× computational super‐resolution and were processed using an Olympus 50× objective lens (NA = 0.8). Scale bars, 150 pixels (b), 30 pixels (c), 40 µm (d), 8 µm (e), 1 cm (f), and 50 µm (g).

Furthermore, we employed the dual‐SLM parallel system, in which phase patterns for generating 3 × 20 × 20 discrete 3D light fields were loaded onto both SLM1 and SLM2. This enabled 2,400 processing points to be generated from two split sub‐pulses derived from one incident pulse. Using an energy of 1 mJ per split incident pulse in each SLM arm and a pulse repetition rate of 10 kHz, this configuration corresponds to a total laser power of 20 W and gives a demonstrated processing throughput of 2,400 processing points pulse^−1^ × 10 000 pulses s^−1^ = 24 000 000 processing points s^−1^ (Figure 4f,g). At a scanning speed of 0.5 m s^−1^, a 5 cm‐long and 50 µm‐wide region can be processed in about 0.1 s. The detailed implementation and results are provided in Figures S15 and S16.

### 2.5 High Manufacturing Flexibility and Broad Material Compatibility

The 3D multi‐foci light‐field shaping and high‐throughput micro/nano manufacturing method not only achieves the aforementioned high fabrication resolution, high processing throughput, and high processing quality but also possesses remarkable manufacturing flexibility and broad material applicability. As shown in Figure 5a, by loading the 3D light field phase pattern onto the SLM, multi‐layer 3D spatial shaping of the light field can be achieved. The specific pattern in Figure 5b illustrates a five‐layer Beijing Institute of Technology emblem fabricated using this manufacturing technology. The overall shape resembles an upright “tree,” with patterns transitioning from a “falcon” at the bottom to a “dove of peace” at the top, as shown in the complete axial distribution diagram in Figure 5c, with a magnified view of the structure presented in Figure 5d.

**FIGURE 5:**
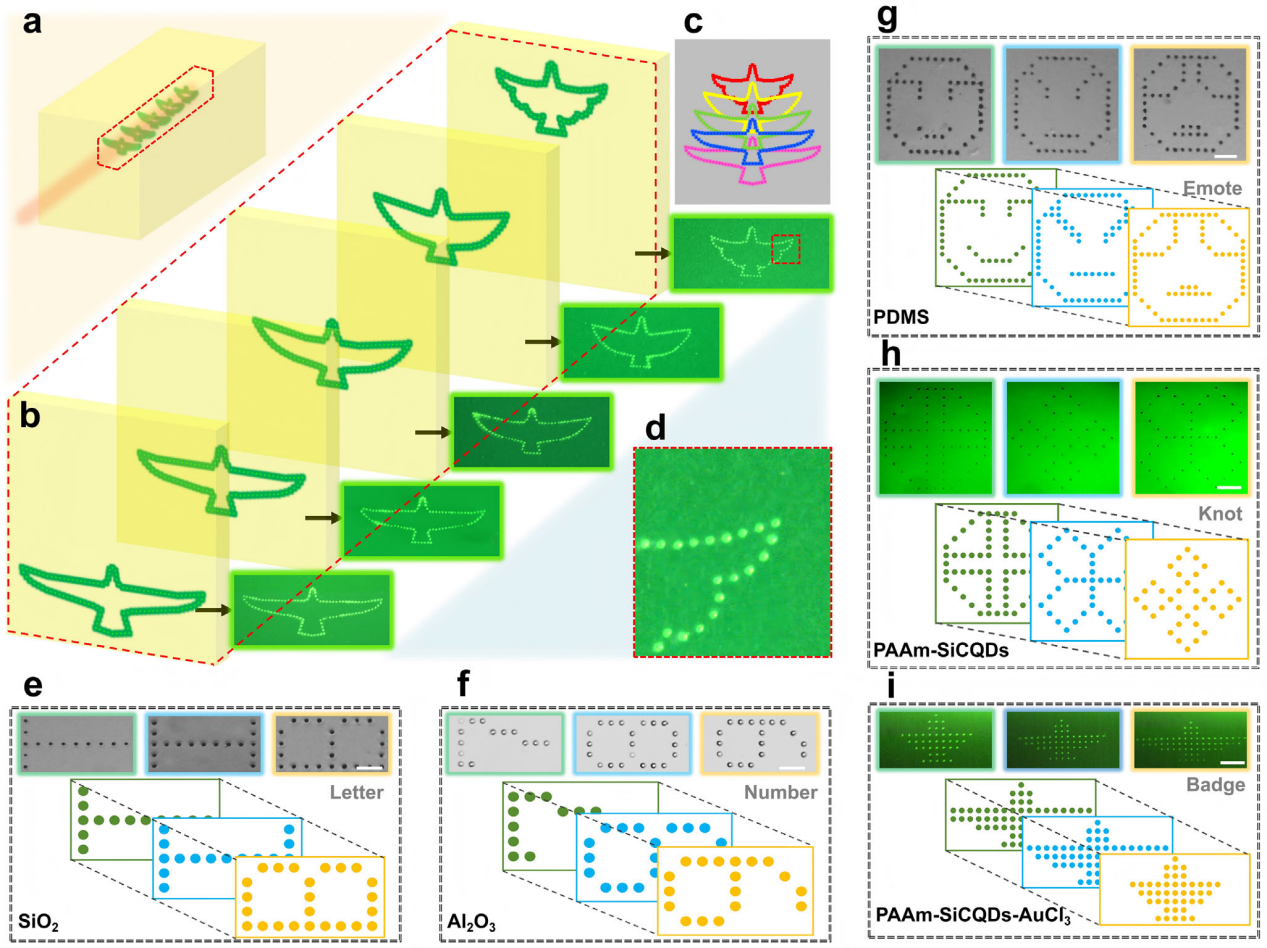
Manufacturing flexibility and broad material compatibility. (a) Three‐dimensional light field for 3D micro/nano manufacturing. (b) Schematic of 3D light‐field display and the corresponding fluorescence image obtained after fabrication. (c) Layered two‐dimensional representation of the 3D “Beijing Institute of Technology” emblem pattern. (d) Zoom‐in view of (b). (e) Letter pattern processed in SiO_2_. (f) Number pattern processed in Al_2_O_3_. (g) Emote pattern processed in PDMS. (h) Knot pattern processed in PAAm‐SiCQDs. (i) Badge pattern processed in PAAm‐SiCQDs‐AuCl_3_. The results in (a–d) were generated using the dual‐SLM parallel system with 3× computational super‐resolution. The results in (e–i) were generated using SLM1 with 3× computational super‐resolution. All processing results were obtained using an Olympus 20× objective lens (NA = 0.45).

Excellent processing results have been achieved across different materials using various patterned light fields. In high‐threshold dielectric materials such as SiO_2_ and Al_2_O_3_ in Figure 5e,f, simple letter and number array patterns have been successfully fabricated. In the flexible organic material polydimethylsiloxane (PDMS) in Figure 5g, more complex patterned structures have been realized. Fluorescence modulation of the patterned dot arrays was achieved in PAAm‐based hydrogels doped with SiCQDs and AuCl_3_, as shown in Figure 5h,i. The corresponding photoluminescence spectra before and after femtosecond laser processing are provided in Figure S17. The effective processing (The design region is processed while the non‐design region remains unprocessed) energy range for different materials is provided in Table S4. The processing results in different materials are shown in Figures S18 and S19 and Table S5. The representative three‐dimensional light field phase patterns are provided in Figure S20.

In PAAm‐SiCQDs, laser processing attenuates the fluorescence intensity of the processed regions, whereas in PAAm‐SiCQDs‐AuCl_3_, laser‐induced fluorescence enhancement is observed. These material‐composition‐dependent fluorescence responses demonstrate that the single‐pulse 3D light‐field manufacturing strategy can be extended from structural patterning to fluorescence‐encoded functional patterning in hydrogel materials, suggesting its potential for optical storage applications. Mechanistic analyses based on X‐ray photoelectron spectroscopy (XPS), transmission electron microscopy (TEM), and molecular dynamics (MD) simulations are provided in Figures S21–S28. All of these experiments demonstrate the high manufacturing flexibility and broad material compatibility of our single‐pulse femtosecond laser 3D multi‐foci light‐field shaping and high‐throughput micro/nano manufacturing method.

## 3 Conclusion

In summary, this work presents a single‐pulse femtosecond‐laser 3D multi‐foci light‐field shaping and high‐throughput manufacturing method that integrates optical spatial shaping with laser micro/nano processing. The proposed multi‐layer noise threshold filtering algorithm suppresses inter‐plane crosstalk by applying region‐prioritized constraints to the defined, interference, and undefined regions, thereby enabling high‐quality 3D light‐field generation. Combined with the multi‐layer over‐sampling algorithm, this method increases the number of laser foci, reduces the effective calculated focal‐spot spacing, and enables single‐pulse 3D fabrication with small fabricated feature size, high throughput, and high processing quality.

In the single‐SLM configuration, a representative 3 × 20 × 20 light field containing 1,200 foci per pulse was experimentally demonstrated. In the dual‐SLM parallel configuration, two split sub‐pulses derived from one incident pulse were used to achieve a demonstrated processing throughput of 24 000 000 processing points s^−1^. The method was further validated in representative inorganic, organic, and hydrogel material systems, including SiO_2_, Al_2_O_3_, PET, PDMS, and PAAm‐based hydrogels. These results demonstrate the manufacturing flexibility and representative material applicability of the proposed single‐pulse femtosecond‐laser 3D multi‐foci light‐field shaping strategy under the tested processing conditions.

Nevertheless, the current implementation still involves trade‐offs among spatial capacity, optical energy utilization, phase‐optimization time, and fabrication uniformity. The generated single‐pulse shaped light field still exhibits a certain degree of nonuniformity, especially because the edge foci are significantly distorted by off‐axis aberrations. In addition, the current high‐throughput processing scheme still relies on static holograms during fabrication, and dynamic switching of arbitrary 3D patterns remains limited by the refresh rate and damage threshold of existing phase‐modulation devices. Therefore, future research should continue to explore light‐field crosstalk control, energy‐utilization feedback, field‐dependent aberration correction, applicability of high‐NA objective lenses and high‐speed phase switching, with the aim of increasing the number of layers and foci while maintaining fabrication quality. Overcoming these limitations will further promote the broader application of single‐pulse 3D light‐field manufacturing technologies.

## 4 Experimental Section

### 4.1 Materials and Sample Preparation

The SiO_2_ and Al_2_O_3_ samples were purchased from Hefei Kejing Materials Technology Co., Ltd. (Hefei, China). Acrylamide (AA, 99%), N,N'‐methylenebisacrylamide (Bis, 99%), ammonium persulfate (APS, 99%), and N,N,N',N'‐tetramethylethylenediamine (TMEDA, 99%) were purchased from Sigma–Aldrich, Inc. (Shanghai, China). Silicon carbide quantum dots (SiCQDs) were purchased from Nanjing Xianfeng Nano Co., Ltd. (Nanjing, China). Gold(III) chloride (AuCl_3_) was purchased from Shanghai Meryer Biochemical Technology Co., Ltd. (Shanghai, China).

To prepare pure polyacrylamide (PAAm), 3 g of acrylamide (AA, 99%), 1.5 mg of N,N'‐methylenebisacrylamide (Bis, 99%), and 20 mg of ammonium persulfate (APS, 99%) were dissolved in 10 mL of deionized water under stirring until completely dissolved. Subsequently, 10 µL of N,N,N',N'‐tetramethylethylenediamine (TMEDA, 99%) was added, and the mixture was stirred for 30 s. The solution was then poured into a mold and allowed to stand for 3 h to ensure complete cross‐linking, yielding pure PAAm. To prepare doped hydrogels, SiCQDs or AuCl_3_ were added to the initial deionized water prior to the addition of monomers, resulting in PAAm‐SiCQDs or PAAm‐SiCQDs‐AuCl_3_, respectively.

### 4.2 Experimental Setup

The experimental setup consisted of a YactoFiber‐FL‐20 ultrafast laser source (Hangzhou Yacto Technology Co., Ltd., Hangzhou, China) with a central wavelength of 1,030 nm, a pulse duration of 250 fs, and a repetition rate of up to 200 kHz. A dual‐SLM parallel system was employed to generate the multi‐layer light field by loading holograms computed via the M‐NTF and M‐OS algorithms. In this configuration, the single pulse was divided into two split sub‐pulses derived from one incident pulse. The system consisted of two SLMs: a PLUTO‐2.1‐NIR‐149 SLM device (HOLOEYE Photonics AG, Berlin, Germany) with a device resolution of 1,920 × 1,080 pixels and a physical pixel pitch of 8 × 8 µm^2^ named SLM1, and a GAEA‐2‐NIR‐069 SLM device (HOLOEYE Photonics AG, Berlin, Germany) with a device resolution of 3,840 × 2,160 pixels and a physical pixel pitch of 3.74 × 3.74 µm^2^ named SLM2. For consistency with SLM1, SLM2 was operated with 2 × 2 pixel binning, corresponding to an effective device resolution of 1,920 × 1,080 pixels and an effective pixel pitch of 7.48 × 7.48 µm^2^. A 4f system, comprising two convex lenses with a focal length of 750 mm, was used to relay the SLM‐modulated wavefront to the objective entrance pupil before laser focusing. The shaped beam was focused using a 20× (NA = 0.45), 50× (NA = 0.8), or 100× (NA = 0.95) microscope objective (Olympus, Tokyo, Japan). A custom three‐axis motion system, incorporating one GTS30V (Newport, USA) and two MLT25 (Newport, USA) electric displacement stages, achieved motion speeds of up to 500 mm s^−1^.

### 4.3 Characterization

The laser processing results of the samples were characterized by a BX51 optical microscope (Olympus, Tokyo, Japan) and a Regulus 8230 scanning electron microscope (Hitachi, Tokyo, Japan). Photoluminescence (PL) spectra were obtained using a micro‐Raman system (Horiba, Kyoto, Japan) with an excitation wavelength of 325 nm and a grating of 600 lines mm^−1^. The TEM images were acquired using a JEM‐2100F transmission electron microscope (JEOL, Tokyo, Japan). X‐ray photoelectron spectroscopy (XPS) measurements were performed using an ESCALAB Xi+ spectrometer (Thermo Fisher Scientific, UK) with a monochromatic Al Kα source (50 µm beam spot).

## Author Contributions

**Shouyu Wu**: conceptualization, investigation, Writing – original draft, Writing – review and editing, visualization, validation, methodology, software, formal analysis, data curation. **Ruiyang Wang**: investigation, writing – original draft, writing – review and editing, methodology, software, formal analysis, data curation. **Lan Jiang**: funding acquisition, project administration, resources, supervision, writing – original draft, writing – review and editing, formal analysis, investigation. **Mengyao Tian**: writing – original draft, writing – review and editing, formal analysis, investigation. **Yongjiu Yuan**: writing – original draft, writing – review and editing, formal analysis, investigation. **Junrui Wu**: writing – original draft, writing – review and editing, validation, formal analysis. **Liang Zhao**: writing – original draft, writing – review and editing, validation, formal analysis. **Ruochen Zhang**: writing – original draft, writing – review and editing, data curation, formal analysis. **Ziqian Ning**: writing – original draft, writing – review and editing, formal analysis, validation, investigation. **Xin Li**: funding acquisition, project administration, resources, supervision, conceptualization, investigation, writing – original draft, writing – review and editing, formal analysis.

## Funding

This research was supported by the National Natural Science Foundation of China (NSFC) (Grant Nos. 52275401 and 52488301).

## Conflicts of Interest

The authors declare no conflicts of interest.

## Supporting information




**Supporting File**: advs77988‐sup‐0001‐SuppMat.docx.

## Data Availability

The data that supports the findings of this study are available in the supplementary material of this article.
