# Supplementary material for: Single‐Pulse Femtosecond Laser 3D Multi‐Foci Light‐Field Engineering via Multi‐Layer Noise Threshold Filtering
Source: Adv Sci (Weinh). 2026 Sep 28:e77988. Online ahead of print. doi: 10.1002/advs.77988 (PMC13618394; doi:10.1002/advs.77988)
Supplement: Supplementary file 1 [file ADVS-9999-e77988-s001.docx]

Supporting Information for:

Single-pulse femtosecond laser 3D multi-foci light-field engineering via multi-layer noise threshold filtering

Shouyu Wu^1^, Ruiyang Wang^1^, Lan Jiang^1,2,3,4^, Mengyao Tian^1^, Yongjiu Yuan^1^, Junrui Wu^1^, Liang Zhao^1^, Ruochen Zhang^1^, Ziqian Ning^1^, Xin Li^1,2,*^

^1^ Laser Micro/Nano-Fabrication Laboratory, School of Mechanical Engineering, Beijing Institute of Technology, Beijing 100081, P. R. China

^2^ Complex and Microscopic Structure Fabrication Technologies National-level Innovation Center, Beijing 100081, P. R. China

^3^ Yangtze Delta Region Academy of Beijing Institute of Technology, Jiaxing 314019, P. R. China

^4^ Beijing Institute of Technology Chongqing Innovation Center, Chongqing 401120, P. R. China

^*^E-mails: [lixin02@bit.edu.cn](mailto:jianglan@bit.edu.cn)

Funding: This research was supported by the National Natural Science Foundation of China (NSFC) (Grant Nos. 52275401 and 52488301).

Keywords: three-dimensional light-field shaping, femtosecond laser, multi-layer noise threshold filtering, multi-layer over-sampling algorithm, micro/nano manufacturing

**Supplementary Note 1. The three-dimensional reconstruction of the single-pulse 3D processing result**

The processing result obtained using the 3D multi-foci light field in the dual-spatial-light-modulator (dual-SLM) parallel system is shown in **Figure S1**. The lower three layers were fabricated by SLM1, whereas the upper three layers were fabricated by SLM2. The axial spacing between adjacent layers was 30 μm.

Notably, the focus spacing of the light fields generated by the two SLMs is slightly different, which is caused by the small difference in pixel pitch between the two SLM devices. In addition, light-field layers located farther from the zero-order light plane tend to undergo stronger transverse shrinkage. To compensate for this depth-dependent shrinkage, a larger preset focal spacing was assigned to layers farther away from the central plane during the light-field design. For the three-layer light fields generated by SLM1 and SLM2, respectively, the designed calculated focal-spot spacings were set to 30, 33, and 36 pixels (3× over-sampling), based on an axial spacing of 30 μm between adjacent layers. This design was used to make the final fabricated structure spacing as uniform as possible.

**Figure S1**. The 3D reconstruction and each layer processing results. Scale bar, 40 μm.

**Supplementary Note 2. The detailed alignment and operation of the dual-SLM parallel system**

The detailed alignment and adjustment procedure of the dual-SLM parallel system is similar to that used for a dual-pulse delayed optical path. First, a blank phase pattern is loaded onto SLM1 while SLM2 is blocked, and the processing position is marked in the processing-imaging software. Then, a blank phase pattern is loaded onto SLM2 while SLM1 is blocked and SLM2 is unblocked. The angular orientation of SLM2 is adjusted until the focal point generated by SLM2 overlaps with that generated by SLM1, thereby achieving alignment in the x–y direction.

The axial alignment in the z direction is expected to be naturally satisfied in principle, because when blank phase patterns are loaded onto both SLM1 and SLM2, the two sub-pulses are not intentionally phase-modulated and propagate through the same common optical path after recombination. Therefore, their zero-order foci should lie at the same axial focal plane after focusing.

After spatial overlap is achieved, the optical path length of the SLM2 arm is adjusted to calibrate the temporal delay between the two sub-pulses. The SLM2 arm is translated until clear interference fringes can be observed after the beam splitter (BS). By scanning the SLM2 position back and forth, the center of the interference-visibility envelope is identified and defined as the temporal zero-delay position. Subsequently, SLM2 is moved 1 mm farther away from the BS, corresponding to a 2 mm round-trip optical-path difference relative to the SLM1 arm.

Because the pulse duration of the femtosecond laser used in this work is 250 fs, the corresponding spatial pulse length is approximately 75 μm. This is much shorter than the introduced 2 mm optical-path difference. Therefore, the three-dimensional light field generated by SLM1 reaches the sample earlier than that generated by SLM2, and the two sub-pulses do not temporally overlap or interfere with each other during processing.

**Supplementary Note 3. Computational benchmark comparison with representative hologram-generation methods**

To further evaluate the performance of the proposed M-NTF + M-OS framework, we performed a computational benchmark comparison with two representative hologram-generation strategies: the direct phase superposition (DPS) method and the mixed-region amplitude freedom (MRAF) method. The DPS method was selected as a representative direct strategy for extending holographic light-field generation to multiple axial planes, whereas MRAF was included as a representative iterative hologram-optimization strategy with good focal uniformity. The proposed M-NTF + M-OS framework was evaluated under the same benchmark framework for comparison.

It should be emphasized that all quantities reported in this Supplementary Note were obtained from numerical calculations or simulated light-field reconstructions. No experimentally measured light-field or fabrication quantities are included in this benchmark.

The five configurations were compared using five computational quantities: hologram calculation time (A), number of generated light-field layers (B), maximum number of generated foci (C), focal-energy nonuniformity (D), and focal-shape nonuniformity (E). The hologram calculation time represents the computational time required to generate the corresponding phase hologram. The number of generated light-field layers and maximum number of generated foci characterize the three-dimensional spatial capacity of the calculated light field. Focal-energy nonuniformity was quantified as the standard deviation of the calculated focal peak energy densities divided by their mean value. Focal-shape nonuniformity was quantified as the standard deviation of the calculated focal-spot FWHM values divided by their mean value. Therefore, smaller values of D and E indicate higher focal-energy and focal-shape uniformity, respectively. The corresponding raw computational results are summarized in **Table S1**.

**Table S1**. Computational benchmark of representative hologram-generation strategies.

| Computational quantity | This work | M-NTF | MRAF + M-OS | MRAF | DPS |
| --- | --- | --- | --- | --- | --- |
| A | 9688 | 576 | 1225 | 75 | 204 |
| B | 3 | 3 | 1 | 1 | 3 |
| C | 1200 | 1200 | 400 | 400 | 300 |
| D | 0.0536 | 0.1317 | 0.0014 | 0.0029 | 0.1532 |
| E | 0.0244 | 0.0516 | 0.0037 | 0.0098 | 0.0724 |

For visualization in **Figure S2**, the five quantities were normalized such that a larger normalized score consistently represents more favorable performance. Larger raw values of B and C indicate better performance because they represent greater three-dimensional spatial capacity. In contrast, smaller raw values of A, D, and E indicate better performance; therefore, the normalization directions of these three quantities were reversed. The radar plot is intended only as a visual summary, whereas the raw values used for the quantitative comparison are provided in Table S1.

**Figure S2.** Computational benchmark comparison with representative hologram-generation methods.

In this computational comparison, conventional MRAF provides favorable focal-energy and focal-shape uniformity with a relatively short hologram calculation time, but the evaluated configuration is limited to two-dimensional light-field optimization. Combining MRAF with M-OS increases the computational sampling density but does not extend the light field to multiple axial planes. DPS can be directly extended to multiple axial planes through phase superposition; however, its simulated multi-layer light fields exhibit relatively large focal-energy and focal-shape variations. M-NTF alone supports multi-layer light-field generation but exhibits lower focal uniformity than the complete framework. By combining multi-layer noise threshold filtering with multi-layer over-sampling, the proposed M-NTF + M-OS framework achieves a more favorable balance between calculated focal quality and three-dimensional spatial capacity.

Overall, this computational benchmark indicates that the principal advantage of the proposed M-NTF + M-OS framework lies in its three-dimensional spatial capacity, particularly the simultaneous generation of multiple light-field layers and a large number of laser foci. This advantage is accompanied by increased hologram calculation time and a trade-off in focal uniformity.

**Supplementary Note 4. Traditional Gerchberg-Saxton (GS) algorithm**

To avoid confusion with equations and symbols used in other Supplementary Notes, all symbols and equations defined in this Supplementary Note are used only within the present section, unless otherwise specified.

The fundamental Gerchberg-Saxton (GS) algorithm employs Fourier iterative cycles. Specifically, the process begins with a Fourier transformation of the time-domain light field, as shown in Equation (1), yielding the computed frequency-domain light field $f^{'}(m,n)$. The phase is then extracted, and the amplitude is replaced by the pre-generated amplitude, as shown in Equation (2). Subsequently, an inverse Fourier transform is applied to the modified frequency-domain light field, as shown in Equation (3), resulting in a computed time-domain light field $\text{F}^{\text{'}}(u,v)$. The phase of this result is then taken, and the amplitude is substituted with the amplitude of the incident light field, as indicated in Equation (4). This cycle continues iteratively until the computed amplitude of $f^{'}(m,n)$, $\text{F}^{\text{'}}(u,v)$ closely approximate the fixed amplitude of $f(m,n)$, $\text{F}(u,v)$, at which point the iteration halts.

Furthermore, using the Fourier transform property at the focal layer of convex lenses, an SLM (spatial light modulator) device loaded with the computed phase of $\text{F}^{\text{'}}(u,v)$, can be placed at the front focal layer of the lens, where a fixed incident amplitude of $\text{F}(u,v)$ is introduced. At the rear focal layer, the pre-generated amplitude of $f(m,n)$ can be obtained, with the computed phase of $f^{'}(m,n)$ omitted here. Through this configuration, a link is established among the fixed incident light field amplitude of $\text{F}(u,v)$, the computed phase of $f^{'}(m,n)$, and the pre-generated amplitude of $f(m,n)$. By projecting a Gaussian laser onto the SLM with the computed phase pattern loaded, it becomes feasible to generate a patterned, single-layer, two-dimensional light field. However, the basic GS algorithm only establishes a connection between one time-domain layer and one frequency-domain layer, limiting its ability to generate a three-dimensional spatial light field.

| $f^{'}(m,n)=\sum_{u=0}^{M-1} \sum_{v=0}^{N-1} F^{'}(u,v)\times\exp(j2\pi(mu/M+nv/N))$ | (1) |
| --- | --- |
| $f^{'}(m,n)=Abs(f(m,n))\times Ang(f^{'}(m,n))$ | (2) |
| $\text{F}^{\text{'}}(u,v)=\sum_{m=0}^{M-1} \sum_{n=0}^{N-1} f^{'}(m,n)\times\exp(-j2\pi(mu/M+nv/N))$ | (3) |
| $F^{'}(u,v)=Abs(F(u,v))\times Ang(F^{'}(u,v))$ | (4) |

Where $f(m,n)$ is the pre-generated light field complex amplitude for the frequency-domain, $f^{'}(m,n)$ is the computed light field complex amplitude for the frequency-domain, $\text{F}(u,v)$ is the incident light field complex amplitude for the time-domain initial layer, $\text{F}^{\text{'}}(u,v)$ is the computed light field for the time-domain initial layer, which is initially set as Gaussian light field distribution. (m,n) denotes the discretized position of the frequency-domain light field, while (u,v) denotes the discretized position of the time-domain light field. *M* and *N* are the upper limits of the light field's discretized dot matrix. *Abs* and *Ang* represent the absolute value and phase value of the data, respectively.

**Supplementary Note 5. Comprehensive lists of nomenclature and equations.**

The equations are provided below and the nomenclature is provided in **Table S2**.

| $Q_{1}=\exp(j2\pi(mu/M+nv/N))$ | (1) |
| --- | --- |
| $f_{x}^{'}(m,n)=\sum_{u=0}^{M-1} \sum_{v=0}^{N-1} (AbsF(u,v)\times(AngF(u,v)+fresnel_{x}))\times Q_{1}$ | (2) |
| $f^{'}(m,n)=Absf^{'}(m,n)\times Angf^{'}(m,n)$ | (3) |
| $Z_{x}=Max(Absf_{x}^{'}(m,n))$ | (4) |
| $Absf_{x}(m,n)=\left\{\begin{matrix} \begin{matrix} \frac{Z_{x}}{Z_{1}}Absf_{x}(m,n) & (m,n)\in A \end{matrix} \\ \begin{matrix} Absf_{x}^{'}(m,n) & \begin{matrix} (m,n)\in B & Absf_{x}^{'}(m,n)<0.5Z_{x} \end{matrix} \end{matrix} \\ \begin{matrix} 0.5Absf_{x}^{'}(m,n) & \begin{matrix} (m,n)\in B & Absf_{x}^{'}(m,n)\geq0.5Z_{x} \end{matrix} \end{matrix} \end{matrix}\right.$ | (5) |
| $Angf_{x}(m,n)=Angf_{x}^{'}(m,n)$ | (6) |
| $f_{x}(m,n)=Absf_{x}(m,n)\times Angf_{x}(m,n)$ | (7) |
| $Q_{2}=\exp(-j2\pi(mu/U+nv/V))$ | (8) |
| $F^{'}(u,v)=\sum_{m=0}^{M-1} \sum_{n=0}^{N-1} f_{x}(m,n)\times Q_{2}-fresnel_{x}$ | (9) |
| $F^{'}(u,v)=AbsF^{'}(u,v)\times AngF^{'}(u,v)$ | (10) |
| $AbsF(u,v)=AbsF(u,v)$ | (11) |
| $AngF(u,v)=AngF^{'}(u,v)$ | (12) |
| $F(u,v)=AbsF(u,v)\times AngF(u,v)$ | (13) |

**Table S2**. Nomenclature

| $F(u,v)$ | distribution of actual incident light field |
| --- | --- |
| $AbsF(u,v)$ | amplitude of actual incident light field |
| $AngF(u,v)$ | phase loaded onto the SLM |
| $F^{'}(u,v)$ | distribution of calculated incident light field |
| $AbsF^{'}(u,v)$ | amplitude of calculated incident light field |
| $AngF^{'}(u,v)$ | phase of calculated incident light field |
| $f_{x}(m,n)$ | distribution of actual pre-generated light field for the *x*-layer |
| $Absf_{x}(m,n)$ | amplitude of actual pre-generated light field for the *x*-layer |
| $Angf_{x}(m,n)$ | phase of actual pre-generated light field for the *x*-layer |
| $f_{x}^{'}(m,n)$ | distribution of calculated pre-generated light field for the *x*-layer |
| $Absf_{x}^{'}(m,n)$ | amplitude of calculated pre-generated light field for the *x*-layer |
| $Angf_{x}^{'}(m,n)$ | phase of calculated pre-generated light field for the *x*-layer |
| $Q_{1}$ | forward discrete Fourier transform operator |
| $Q_{2}$ | inverse discrete Fourier transform operator |
| $fresnel_{x}$ | Fresnel phase of pre-generated light field for the *x*-layer |
| $Z_{x}$ | the maximum amplitude of pre-generated light field for the *x*-layer |
| $A$ | the patterned area of the pre-generated light field |
| $B$ | the noise area of the pre-generated light field |

**Supplementary Note 6. The algorithm logic of multi-layer noise threshold filtering.**

Step 1. The Fresnel phase of the pre-generated light field for the *1st*-layer $fresnel_{1}$ is added to the actual incident light field. Then the forward Fourier transform is applied in order to obtain the distribution of calculated pre-generated light field for the 1st-layer $f_{1}^{'}(m,n)$ as shown in equation (1), (2). The distribution of this light field $f_{1}^{'}(m,n)$ is constituted of its amplitude $Absf_{1}^{'}(m,n)$ and phase $Angf_{1}^{'}(m,n)$, as shown in equation (3). For its amplitude, the maximum value $Z_{1}$ is extracted as an indicator of the amplitude for this layer as shown in equation (4).

Step 2. Based on the calculated pre-generated light field $f_{1}^{'}(m,n)$, the actual pre-generated light field $f_{1}(m,n)$ is replaced, as shown in equation (5), (6). Specifically, the amplitude in the patterned area $A$ is replaced with the actual pre-generated light field amplitude $Absf_{1}(m,n)$ for the *1st*-layer to strictly constrain the processing area. In the noise area *B*, if the amplitude of calculated pre-generated light field $Absf_{1}^{'}(m,n)$ is below the filtering threshold, no replacement occurs; if the amplitude exceeds the threshold, it is halved and replaced, reducing the noise to below the threshold. The actual pre-generated light field for the *1st*-layer $f_{1}(m,n)$ has been updated (equation (7)).

Step 3. The inverse Fourier transform is applied in order to obtain the distribution of calculated incident light field $F^{'}(u,v)$ using equations (8), (9). The distribution of this light field $F^{'}(u,v)$ is constituted of its amplitude $AbsF^{'}(u,v)$ and phase $AngF^{'}(u,v)$, as shown in equation (10).

Step 4. The phase loaded onto the SLM $AngF(u,v)$ is replaced by the phase of calculated incident light field $AngF^{'}(u,v)$,and the amplitude of actual incident light field is retained, as shown in equation (11), (12). The actual incident light field has been updated (equation (13)).

Step 5. Repeat Steps 1–4 using the Fresnel phases of the pre-generated light fields for the second and third layers.

Step 6. Repeat Steps 1–5 until the calculated amplitudes of all pre-generated light-field layers converge to the target amplitudes. Then, output the final phase pattern $AngF(u,v)$.

**Supplementary Note 7. Quantitative evaluation of M-NTF for in-plane uniformity and inter-plane crosstalk suppression**

The purpose of the M-NTF algorithm is not to minimize the background noise over the entire reconstructed plane. Instead, it redistributes the optimization priority according to the processing relevance of different regions. Specifically, the defined region, corresponding to the target processing pattern, is strictly constrained to a relative amplitude of 1 to ensure a high and uniform intensity distribution for high-quality fabrication. The interference region, located adjacent to the defined region, is strictly constrained to a relative amplitude of 0. This region acts as a protective buffer that prevents neighboring in-plane crosstalk from disturbing the defined region. By contrast, the undefined region is assigned a lower constraint priority. Residual noise in this region is acceptable as long as its intensity remains below the material processing threshold and therefore does not contribute to effective fabrication.

As shown in **Figure S3**, the reconstructed plane with M-NTF may contain more visible residual background noise in the undefined region than the result without M-NTF. However, the intensity profile in the defined pattern region is more uniform, which is more relevant to the final fabrication quality. This result reflects the design principle of M-NTF: rather than enforcing excessive global noise suppression, the algorithm prioritizes the intensity uniformity of the defined region and the suppression of the interference region, thereby improving the quality of the fabricated structures.

For the inter-plane analysis, a single-pulse Gaussian beam was shaped into a three-layer dot-matrix light field containing the letters B, I, and T at different axial positions. The intensity distributions in the defined and interference regions with and without M-NTF are shown in **Figure S4**, **S5**. With M-NTF, the normalized intensity in the defined pattern regions remains above 90%, while the residual intensity in the interference regions is suppressed below 10%. In contrast, without M-NTF, the defined pattern regions show reduced intensity uniformity and the residual interference in non-target regions can exceed 20%. These results demonstrate that M-NTF effectively preserves the target-pattern intensity while suppressing inter-plane crosstalk in the interference regions to below the processing-relevant threshold.

**Figure S3**. In-plane energy distribution of the single-pulse three-dimensional light field with and without M-NTF. (a) Reconstructed in-plane light-field distribution with M-NTF. (b) Normalized intensity profile extracted along the dashed line in Figure S3a. (c) Reconstructed in-plane light-field distribution without M-NTF. (d) Normalized intensity profile extracted along the dashed line in Figure S3c. Although residual background noise is more visible in the undefined region with M-NTF, the defined region exhibits improved intensity uniformity because the optimization priority is concentrated on the defined region and the adjacent interference region, which are most relevant to fabrication quality.

**Figure S4**. Inter-plane intensity distribution in the defined regions of a three-layer B/I/T light field. (a) Schematic of the three-layer dot-matrix light-field design, where the letters B, I, and T are located at different axial positions. (b) Inter-plane light-field distribution with M-NTF. (c) Inter-plane light-field distribution without M-NTF. (d-f) Normalized intensity profiles along the left, middle, and right dashed lines in Figure S4b. (g-i) Normalized intensity profiles along the left, middle, and right dashed lines in Figure S4c.

**Figure S5**. Inter-plane intensity distribution in the interference regions of a three-layer B/I/T light field. (a) Schematic of the three-layer dot-matrix light-field design, where the letters B, I, and T are located at different axial positions. (b) Inter-plane light-field distribution with M-NTF. (c) Inter-plane light-field distribution without M-NTF. (d–f) Normalized intensity profiles along the left, middle, and right dashed lines in Figure S5b. (g–i) Normalized intensity profiles along the left, middle, and right dashed lines in Figure S5c.

**Supplementary Note 8. The detailed calculation formula for over-sampling algorithm**

To avoid confusion with equations and symbols used in other Supplementary Notes, all symbols and equations defined in this Supplementary Note are used only within the present section, unless otherwise specified.

For the present optical system, the incident light field on the SLM plane and the pre-generated light field at the Fourier plane are related through a two-dimensional Fourier transform. The Fourier lens maps the spatial frequency $f_{x}$ of the incident light field to the physical coordinate $x_{f}$ in the pre-generated light-field plane, as described by Equation (1). Accordingly, the sampling interval $\Delta x_{f}$ in the pre-generated light-field plane satisfies Equation (2).

For the discrete Fourier transform, the spatial-frequency sampling interval $\Delta f_{x}$ of the incident light field is determined by the SLM sampling interval and the computational grid size, as given by Equation (3). Therefore, the relationship between the sampling interval $\Delta x_{f}$ in the pre-generated light-field plane and the SLM pixel pitch can be obtained as Equation (4).

| $x_{f}=\lambda f\cdot f_{x}$ | (1) |
| --- | --- |
| $\Delta x_{f}=\lambda f\cdot\Delta f_{x}$ | (2) |
| $\Delta f_{x}=\frac{1}{N\Delta x_{\text{SLM}}}$ | (3) |
| $\Delta f_{x}=\frac{\lambda f}{N\Delta x_{\text{SLM}}}$ | (4) |

Here, $x_{f}$ is the physical coordinate in the pre-generated light-field plane, $f_{x}$ is the spatial frequency of the incident light field, $\Delta x_{f}$ is the sampling interval in the pre-generated light-field plane, $\Delta f_{x}$ is the spatial-frequency sampling interval of the incident light field, $\Delta x_{\text{SLM}}$ is the SLM sampling interval, or equivalently the SLM pixel pitch, $\lambda$ is the laser wavelength, $f$ is the focal length of the Fourier lens, and $N$ is the number of sampling pixels along one transverse direction of the computational grid.

For the computational conventional-resolution light field, substituting $\lambda$=1,030 nm, $f$=750 mm, $N$= 1,080, and $\Delta x_{\text{SLM}}$=8 μm gives $\Delta x_{f}$=89.4 μm. For the 3× computational super-resolution light field, $N$ is increased to 3,240 while the other parameters remain unchanged, resulting in $\Delta x_{f}$= 29.8 μm.

**Supplementary Note 9. Comparison of light field distribution at different over-sampling factors**

Different over-sampling factors correspond to different computational super-resolution magnifications. Without over-sampling, each focus exhibits a flat-top profile and occupies a 2 × 2-pixel area. As the over-sampling factor increases, the shape of the foci approaches a Gaussian shape asymptotically (**Figure S6**). In principle, super-resolution calculations can be performed at higher over-sampling factors, provided the computational complexity is manageable. However, the practically achievable precision is ultimately constrained by the optical aperture, phase aberrations, pulse energy, and material response.

**Figure S6**. Comparison of the light field at different over-sampling magnifications.

**Supplementary Note 10. Effect of calculated focal-spot size and spacing**

The choice of different dot matrix focus sizes significantly impacts the final structural formation. We compared basic dot matrix focus sizes of 1 × 1, 2 × 2, 3 × 3, and 4 × 4 pixels as shown in **Figure S7**, and found that even-numbered calculated focal-spot size like 2 × 2 and 4 × 4 achieve better uniformity in intensity distribution compared to odd-numbered focus sizes like 1 × 1 and 3 × 3. We believe this phenomenon is due to the inherent characteristics of Fourier transformation. Considering factors such as the algorithm convergence speed, fabrication precision, and phase utilization, we ultimately determined that a 2 × 2 pixel calculated focal-spot size is the most suitable basic calculated focal-spot size.

Calculated focal-spot spacing is a critical metric for controlling pattern resolution and uniformity. Smaller calculated focal-spot spacing leads to poorer pattern uniformity, increased computational complexity, and higher demands on algorithm convergence. Larger calculated focal-spot spacing results in fewer dot-matrix elements, but facilitates the generation of high-quality light field.

**Figure S7**. The light field distribution of different calculated focal-spot size and spacing.

**Supplementary Note 11. Comparative analysis of calculated light fields, measured light fields, and processing results in SiO_2_ with different over-sampling factors**

To evaluate the influence of the over-sampling factor, three groups of comparative experiments were performed, as shown in Figure S8. Group 1 corresponds to no over-sampling (**Figure S8a**), Group 2 corresponds to 3× over-sampling (**Figure S8b**), and Group 3 corresponds to 9× over-sampling (**Figure S8c**).

The measured light-field distributions in Figure S8 were characterized using a beam quality analyzer positioned between M3 and M4 in the optical path shown in Figure 1a, before the focusing objective. Therefore, these measurements characterize the pre-objective multi-foci intensity distributions and do not constitute direct measurements of the final high-NA focal PSF at the sample plane.

The statistical results show that increasing the over-sampling factor can maintain the uniformity of the light-field energy distribution (**Figure S9a, d**), while reducing the generated calculated focal-spot size and improving the uniformity (**Figure S9c, f**). This further enables the fabrication of structures with higher uniformity and smaller feature size (**Figure S9g**). For the 9× over-sampling condition, the repeated measurements yielded a mean fabricated feature size of 94.06 ± 24.02 nm, with a minimum measured value of 58.75 nm within the repeated-measurement dataset. However, this improvement is achieved at the cost of reduced energy utilization efficiency (**Figure S9b, e, h**) and a longer phase-optimization time (**Figure S9i**).

**Figure S8.** Demonstration of calculated light fields, measured light fields, and fabrication results with different over-sampling factors. (a) Calculated, measured light fields and processing results without over-sampling. (b) Calculated, measured light fields and processing results with 3× over-sampling. (c) Calculated, measured light fields and processing results with 9× over-sampling. Scale bars 2 μm.

**Figure S9**. Comparative results of calculated light fields, measured light fields, and fabrication results under different over-sampling factors. (a) Normalized calculated peak intensity of Group 1-3. (b) Calculated equivalent energy efficiency of Group 1-3. (c) Calculated focal-spot FWHM of Group 1-3. (d) Normalized measured peak intensity of Group 1-3. (e) Measured absolute diffraction efficiency of Group 1-3. (f) Measured focal-spot FWHM of Group 1-3. (g) Fabricated structure feature size of Group 1-3. (h) Measured equivalent energy efficiency of Group 1-3. (i) Phase-optimization time of Group 1-3.

In Figure S9, the calculated equivalent energy efficiency is defined as the calculated peak intensity of the light field multiplied by the number of foci, divided by the calculated peak intensity obtained when a blank phase is loaded. The measured equivalent energy efficiency is defined as the measured minimum single-pulse energy required for complete fabrication when a blank phase is loaded, divided by the measured minimum single-pulse energy per focus required for complete fabrication with the multi-foci phase pattern. The measured absolute diffraction efficiency is defined as the ratio of the measured laser energy after the SLM to that before the SLM. The feature size was defined as the major-axis length of the fitted ellipse obtained by fitting the boundary of each fabricated structure in the microscopy images. All phase-pattern optimization times were measured as wall-clock times for a single hologram calculation on the same workstation equipped with an Intel Core i5-13600K CPU and an NVIDIA GeForce RTX 3060 GPU.

**Supplementary Note 12. Three-dimensional light-field energy distributions for different numbers of layers**

The light field energy distribution for different numbers of layers using a single pulse by SLM1 is shown in **Figure S10**. It is evident that the single-layer light field exhibits a maximum of 1,600 laser foci, the three-layer light field exhibits a maximum of 2,700 laser foci, the five-layer light field exhibits a maximum of 500 laser foci, and the ten-layer light field exhibits a maximum of 100 laser foci.

**Figure S10**. Light-field energy distributions for different numbers of layers. (a) The single-layer light field energy distribution with calculated focal-spot spacing of 10 pixels. (b) The three-layer light field energy distribution with calculated focal-spot spacing of 30 pixels. (c) The five-layer light field energy distribution with calculated focal-spot spacing of 90 pixels. (d) The ten-layer light field energy distribution with calculated focal-spot spacing of 90 pixels. (e) Zoom-in view of (a). (f) Zoom-in view of (b). (g) Zoom-in view of (c). (h) Zoom-in view of (d).

**Supplementary Note 13. Comparative analysis of calculated light fields, measured light fields, and processing results in PET for different light fields**

To evaluate the influence of the over-sampling factor, six groups of comparative experiments were performed, as shown in **Figure S11, S12**. Group 4 corresponds to 3 × 4 × 4 light field without over-sampling (**Figure S11a**), Group 5 corresponds to 3 × 10 × 10 light field without over-sampling (**Figure S11b**), Group 6 corresponds to 3 × 20 × 20 light field without over-sampling (**Figure S11c**), Group 7 corresponds to 3 × 4 × 4 light field with 3× over-sampling (**Figure S12a**), Group 8 corresponds to 3 × 10 × 10 light field with 3× over-sampling (**Figure S12b**), Group 9 corresponds to 3 × 20 × 20 light field with 3× over-sampling (**Figure S12c**).

The statistical results show that adding over-sampling can improve the uniformity of the light-field energy distribution (**Figure S13a, d**), while reducing the generated calculated focal-spot size and improving the uniformity (**Figure S13c, f**). This further enables the fabrication of structures with higher uniformity (**Figure S13g**). Notably, compared with the results in **Supplementary Note 11**, the over-sampling strategy can also improve the energy utilization efficiency when the calculated focal-spot size is not intentionally compressed (**Figure S13b, e, h**). Nevertheless, this improvement is still accompanied by a substantial increase in the phase-optimization time (**Figure S13i**).

The measured light-field distributions in Figure S11 and S12 were obtained at the same pre-objective measurement plane described in Supplementary Note 11.

It should also be noted that, as the processing field and the number of foci increase, edge foci begin to exhibit noticeable distortion, as can be directly observed from the measured light-field and fabrication results in Groups 6 and 9 (Figure S11, S12). This distortion is mainly attributed to the off-axis aberration of the plano-convex lenses (L1). When the relative position between the light field and the plano-convex lenses is shifted, the distortion consistently becomes more pronounced for foci farther away from the optical axis.

**Figure S11.** Demonstration of calculated light fields, measured light fields, and processing results under different light fields without over-sampling. (a) Calculated, measured light fields and processing results for the 3 × 4 × 4 light field. (b) Calculated, measured light fields and processing results for the 3 × 10 × 10 light field. (c) Calculated, measured light fields and processing results for the 3 × 20 × 20 light field. Scale bars 40 μm.

**Figure S12.** Demonstration of calculated light fields, measured light fields, and processing results under different light fields with 3× over-sampling. (a) Calculated, measured light fields and processing results for the 3 × 4 × 4 light field. (b) Calculated, measured light fields and processing results for the 3 × 10 × 10 light field. (c) Calculated, measured light fields and processing results for the 3 × 20 × 20 light field. Scale bars 40 μm.

**Figure S13**. Comparative results of calculated light fields, measured light fields, and fabrication results for different light fields. (a) Normalized calculated peak intensity of Group 4-9. (b) Calculated equivalent energy efficiency of Group 4-9. (c) Calculated focal-spot FWHM of Group 4-9. (d) Normalized measured peak intensity of Group 4-9. (e) Measured absolute diffraction efficiency of Group 4-9. (f) Measured focal-spot FWHM of Group 4-9. (g) Fabricated structure feature size of Group 4-9. (h) Measured equivalent energy efficiency of Group 4-9. (i) Phase-optimization time of Group 4-9.

In Figure S13, the calculated equivalent energy efficiency is defined as the calculated peak intensity of the light field multiplied by the number of foci, divided by the calculated peak intensity obtained when a blank phase is loaded. The measured equivalent energy efficiency is defined as the measured minimum single-pulse energy required for complete fabrication when a blank phase is loaded, divided by the measured minimum single-pulse energy per focus required for complete fabrication with the multi-foci phase pattern. The measured absolute diffraction efficiency is defined as the ratio of the measured laser energy after the SLM to that before the SLM. The feature size was defined as the major-axis length of the fitted ellipse obtained by fitting the boundary of each fabricated structure in the microscopy images. All phase-pattern optimization times were measured as wall-clock times for a single hologram calculation on the same workstation equipped with an Intel Core i5-13600K CPU and an NVIDIA GeForce RTX 3060 GPU.

**Supplementary Note 14. Aberration correction for three-dimensional light-field processing**

To avoid confusion with equations and symbols used in other Supplementary Notes, all symbols and equations defined in this Supplementary Note are used only within the present section, unless otherwise specified.

In practical single-pulse three-dimensional processing, the refractive-index mismatch between air and the transparent materials can introduce depth-dependent optical aberration. This effect becomes more pronounced as the focal depth inside the material increases, leading to focal elongation and reduced peak intensity. Therefore, an aberration-correction phase was introduced according to the refractive index of the material and the processing depth.

Based on the generated phase pattern of the three-dimensional light field, we introduced an additional correction phase term to compensate the aberration of the entire 3D light field, as shown in Equation (1). The correction phase is mainly determined by the optical path difference at different effective pupil positions, as described in Equation (2). By substituting Equations (3)–(5) into Equation (2), the optical path difference can be expressed as a function only of the normalized pupil coordinate $\text{ρ}$. Since the SLM plane is relayed to the effective pupil plane of the objective through the 4f optical system, further substituting this result into Equation (6) gives the correction phase distribution corresponding to each SLM position. In addition, refractive indices for different materials used for aberration correction are shown in **Table S3**.

$$\text{AngF}_{\text{final}}\text{(}\text{u}\text{,}\text{v}\text{)}\text{ }\text{=}\text{ }\text{mod}\left[ \text{AngF}\text{(}\text{u}\text{,}\text{v}\text{)+}{\text{Ang}\text{F}}_{\text{coff}}\text{(}\text{u}\text{,}\text{v}\text{),}\text{ }\text{2π} \right]$$

(1)

$$\text{AngF}_{\text{coff}}\text{(}\text{u}\text{,}\text{v}\text{)=}\text{−}\frac{\text{2}\text{π}}{\text{λ}}\text{OPD}\text{(}\text{ρ}\text{,}\text{z}\text{)}$$

(2)

$$\text{OPD}\text{(}\text{ρ}\text{,}\text{z}\text{)}\text{=}\text{z}\left[ \text{n}_{\text{2}}\cos\text{θ}_{\text{2}}\text{−}\text{n}_{\text{1}}\cos\text{θ}_{\text{1}}\text{−}\left( \text{n}_{\text{2}}\text{−}\text{n}_{\text{1}} \right) \right]$$

(3)

$$\text{n}_{\text{2}}\text{sin} \text{θ}_{\text{2}}\text{=}\text{n}_{\text{1}}\sin\text{θ}_{\text{1}}$$

(4)

$$\sin\text{θ}_{\text{1}}\text{=}\frac{\text{NA}}{\text{n}_{\text{1}}}\text{ρ}$$

(5)

$$\text{ρ}\text{=}\frac{\sqrt{{\text{(}\text{u}\text{−}\text{u}_{\text{0}}\text{)}}^{\text{2}}\text{+}{\text{(}\text{v}\text{−}\text{v}_{\text{0}}\text{)}}^{\text{2}}}}{\text{R}_{\text{p}}}$$

(6)

Here, $\text{AngF}_{\text{final}}$ is the final corrected phase loaded onto the SLM, $\text{AngF}$ is the phase loaded onto the SLM without correction,$\text{ }\text{AngF}_{\text{coff}}$ is the corrected phase, $\text{λ}$ is the laser wavelength, $\text{OPD}$ is the optical path difference, $\text{θ}_{\text{1}}$ and $\text{θ}_{\text{2}}$ are the ray angles before and after refraction, $\text{n}_{\text{1}}$ and $\text{n}_{\text{2}}$ are the refractive indices of air and the workpiece material, respectively, $\text{z}$ is the focal depth inside the material, $\text{NA}$ is the numerical aperture of the objective, $\text{ρ}$ is the normalized pupil coordinate.

**Table S3**. Refractive indices used for aberration correction.

| Material | Refractive index |
| --- | --- |
| Air | 1.00 |
| PET | 1.57 |
| PDMS | 1.41 |
| PAAm | 1.35 |
| SiO_2_ | 1.45 |
| Al_2_O_3_ | 1.75 |

The refractive indices listed here were used for the aberration-correction calculation at the laser wavelength of 1,030 nm. For polymeric and hydrogel materials, the values should be regarded as approximate effective refractive indices because they can vary with composition, water content, curing condition, and molecular orientation.

To clearly demonstrate the effect of aberration correction, four-side-polished SiO_2_ was selected as a representative material, which allowed the fabricated structures to be observed from both the x-z and x-y directions. The corresponding fabrication results are shown in Figure S14. First, for near-surface (within a depth of 300 μm) processing, good multi-layer fabrication could still be achieved without aberration correction. Second, without aberration correction, as the processing depth increased, the refractive-index mismatch caused axial elongation of the focal spots and a gradual decrease in the energy density, eventually leading to fabrication failure. After applying the aberration-correction phase, the fabrication capability at larger depths could be recovered. Third, under the current conditions with a limited number of layers and a relatively small interlayer spacing, a single correction phase applied to the entire 3D light field was sufficient, and layer-specific correction was not required. For different materials, the corresponding correction phase can be superimposed according to the refractive index of each material.

Figure S14. Comparison of fabrication results before and after aberration correction in four-side-polished SiO_2_.

**Supplementary Note 15. Demonstration of repeatability of high-throughput processing results.**

To further validate the experimentally demonstrated processing throughput of 24,000,000 processing points s^−1^, repeated high-throughput fabrication experiments were performed, as shown in Figure S15. The fabricated structures were evaluated in terms of fabrication yield, feature-size variation, and repeatability among independent experiments.

For Group 1 fabricated using a 50× objective lens with NA = 0.8, a small number of abnormal structures, including locally connected features and noise-induced structures, were observed. These abnormal structures were regarded as fabrication failures in the yield analysis. In contrast, the 10 × 10 fabricated structures in Group 2 showed a fabrication yield of 100% in the repeated experiments. The corresponding statistical analysis of the fabricated feature size and fabrication yield is summarized in Figure S16.

The results from three independent fabrication experiments show that the mean feature size and fabrication yield remain stable among repeated trials. This indicates good repeatability of the high-throughput fabrication process.

**Figure S15.** Demonstration of repeated high-throughput fabrication results.

**Figure S16.** Statistical analysis of repeated high-throughput fabrication results, including fabricated feature size, feature-size variation, fabrication yield, and repeatability among independent experiments.

**Supplementary Note 16. Fluorescence modulation of the patterned dot arrays.**

The purchased silicon carbide quantum dots (SiCQDs) inherently exhibit strong photoluminescence, which is retained in the as-prepared PAAm-SiCQDs composite. After femtosecond laser processing, the fluorescence intensity of the processed regions in PAAm-SiCQDs decreases significantly, as shown in Figure 5h and **Figure S17a**. This attenuation is associated with laser-induced modification of the PAAm matrix and reduced photoluminescence of the SiCQDs.

In a separate AuCl_3_-containing PAAm-SiCQDs composite, laser processing led to fluorescence enhancement rather than attenuation, as shown in Figure 5i and **Figure S17b**. This enhancement is likely associated with laser-induced reduction of AuCl_3_ into Au-containing nanoparticles, which may contribute to metal-enhanced fluorescence of SiCQDs. These results demonstrate composition-dependent fluorescence attenuation or enhancement in different PAAm-based hydrogel composites.

**Figure S17**. Photoluminescence response of PAAm-based hydrogel composites before and after femtosecond laser processing. (a) Photoluminescence spectra of PAAm-SiCQDs before and after processing. (b) Photoluminescence spectra of PAAm-SiCQDs-AuCl_3_ before and after processing.

**Supplementary Note 17. The effective processing energy range for different materials**

The materials investigated in this work, including inorganic high-threshold materials, soft polymers, and hydrogel composite systems, have very different nonlinear absorption and modification thresholds. Therefore, we selected three representative materials and performed threshold calibration using the same three-dimensional light field. We defined *E_L_* as the minimum pulse energy at which all designed foci can be successfully processed, and *E_H_* as the minimum pulse energy at which unintended processing spots begin to appear. The corresponding values are summarized in Table S4.

**Table S4**. Processing energy window calibration for representative materials

| Material (light field) | *E_L_* (μJ) | *E_H_* (μJ) | Ratio (*E_H_*/*E_L_*) |
| --- | --- | --- | --- |
| PET (3 × 4 × 4, 3× over-sampling) | 29.2 | 61.9 | 2.12 |
| PDMS (3 × 4 × 4, 3× over-sampling) | 66.7 | 144.1 | 2.16 |
| SiO_2_ (3 × 4 × 4, 3× over-sampling) | 119.8 | 266.1 | 2.22 |
| PET (3 × 10 × 10, 3× over-sampling) | 186.4 | 344.8 | 1.85 |
| PDMS (3 × 10 × 10, 3× over-sampling) | 413.8 | 773.8 | 1.87 |
| SiO_2_ (3 × 10 × 10, 3× over-sampling) | 769.3 | >1000 | / |
| PET (3 × 20 × 20, 3× over-sampling) | 734.5 | >1000 | / |

The results show that, although the absolute processing threshold varies among different materials, the ratio between *E_H_* and *E_L_* remains relatively consistent for the same light field. For higher-threshold materials, the absolute width of the effective processing energy window is larger. However, in practical experiments, the laser pulse energy is generally adjusted by an attenuation wheel in a proportional manner rather than by an absolute energy offset. Therefore, the usable processing window in terms of relative attenuation is similar among these materials.

It should be noted that, according to the ideal calculation, *E_H_* would be expected to be approximately four times *E_L_*, because the relative energy density in the undefined region is below 0.25. In practice, however, the experimentally measured ratio is much smaller than this ideal value. This is mainly because the calculated light field itself has a certain degree of nonuniformity, which can be further amplified by the optical system, as shown in **Supplementary Note 13**.

**Supplementary Note 18. The processing results in different materials**

To evaluate the processing uniformity and stability, we performed three independent fabrication experiments in PET, PDMS, and SiO₂ using 3 × 20 × 20, 3 × 10 × 10, and 3 × 5 × 5 laser fields, respectively. To facilitate optical characterization and statistical measurement of the fabricated feature diameters, the uppermost light-field layer was positioned at the material surface. **Figure S18** shows the processing results from three independent experiments in different materials.

To evaluate the uniformity of each individual fabrication result, we measured the diameters of all fabricated features along the diagonal of each processed array. The diagonal was selected because it covers both the central and edge regions of the light field, thereby reflecting position-dependent nonuniformity within the processing area. For each fabricated feature, the maximum lateral diameter was used for statistical analysis, so that focal elongation and shape distortion could be reflected as sensitively as possible. **Figure S19** shows the fabricated feature diameters obtained from each individual fabrication result.

Finally, the processing results from different independent experiments, including the mean value, standard deviation, and coefficient of variation, are listed in **Table S5**. It can be seen that, for the same light field, the coefficient of variation of the fabricated features shows only small differences among different fabrication runs. In contrast, the coefficient of variation differs more significantly among different light fields, and it decreases markedly as the number of foci is reduced.

**Figure S18**. The processing results from the three independent experiments in different materials. Scale bars, 16 μm.

**Figure S19.** The fabricated feature diameters obtained from each individual fabrication result.

**Table S5**. The processing results from different independent experiments.

| Group | Mean diameter [μm] | Standard deviation [μm] | Coefficient of variation [%] |
| --- | --- | --- | --- |
| a1 | 2.97 | 0.40 | 13.46 |
| a2 | 3.09 | 0.53 | 17.10 |
| a3 | 3.24 | 0.60 | 18.60 |
| b1 | 3.44 | 0.34 | 9.97 |
| b2 | 3.32 | 0.38 | 11.33 |
| b3 | 3.30 | 0.36 | 11.00 |
| c1 | 3.18 | 0.15 | 4.75 |
| c2 | 3.12 | 0.12 | 3.80 |
| c3 | 3.15 | 0.09 | 2.87 |

**Supplementary Note 19. The representative three-dimensional light field phase patterns**

The phase patterns corresponding to the representative 3 × 30 × 30 shaped light field and the 3 × 20 × 20, 3 × 10 × 10, and 3 × 5 × 5 processing light fields are provided in **Figure S20**. When these phase patterns are loaded onto either SLM at the same scale and used with the optical configuration described in this work, the corresponding three-dimensional light fields can be generated in the optical setup.

**Figure S20**. The representative three-dimensional light field phase patterns.

**Supplementary Note 20. Mechanistic analysis of laser-induced fluorescence modulation in PAAm-based hydrogel composites**

Supplementary Note 20 provides a mechanistic analysis of laser-induced fluorescence modulation in PAAm-based hydrogel composites (**Figure S21**) using XPS, TEM, and molecular dynamics simulations. These analyses are intended to provide qualitative insight into possible laser-induced structural evolution rather than to fully reproduce the complete femtosecond laser–matter interaction process.

**Figure S21**. Proposed laser-induced structural evolution in the PAAm-SiCQDs material system.

Based on the elemental proportions determined from the XPS survey spectrum of the PAAm-SiCQDs material system, as shown in **Figure S22**, a molecular model composed of multiple acrylamide chains was constructed. The simulated system used periodic boundary conditions in the x, y, and z directions and was evolved under an NVT ensemble with a Langevin thermostat. The simulation was integrated using the Verlet algorithm with a time step of 0.1 fs. A PAAm molecular model consisting of 1,992 atoms was constructed to represent the microscopic structure of the experimental sample, as shown in **Figure S23**. The ReaxFF reactive force field was used to describe possible bond breaking, bond formation, cross-linking, and chemical reactions during laser-induced heating.

**Figure S22**. XPS survey spectrum of the initial PAAm-SiCQDs material system.

**Figure S23**. Initial model of the PAAm-SiCQDs material system.

**Figure S24**. Simulation results of the PAAm-SiCQDs material system at different temperatures.

The simulation results at different temperatures and delay times are shown in **Figure S24**. At the initial stage of laser-induced heating, the internal structure remains relatively intact. After continued heating, chain-segment breakage, local structural rearrangement around SiCQDs, and the formation of small molecular fragments, such as CO and H_2_, can be observed. These results suggest that laser processing may induce polymer-chain cleavage and carbonization-related structural evolution in the PAAm-SiCQDs composite.

To further analyze the compositional changes, the C/O-related bonding evolution during heating was extracted from the simulation results, as shown in **Figure S25**. The increase in the C:O ratio is consistent with the XPS survey spectrum after laser processing, as shown in **Figure S26**. In the high-resolution C 1s spectrum, the C–C component increases after processing, whereas the C=O component decreases, as shown in **Figure S27**. This suggests deoxygenation and carbonization/partial graphitization of the PAAm-SiCQDs material system during laser processing. The decrease in the C=O-related signal in the O 1s spectrum is also consistent with this interpretation.

**Figure S25**. C:O ratio changes in PAAm/SiCQDs under different femtosecond laser parameters.

**Figure S26**. XPS survey spectrum of the PAAm-SiCQDs material system after laser processing.

**Figure S27**. XPS high-resolution spectrum of the PAAm-SiCQDs material system.

In addition, the apparent size increase or aggregation of SiCQDs observed by TEM, as shown in **Figure S28**, together with the local atomic enrichment around the quantum dots suggested by molecular dynamics simulations, may contribute to the reduced photoluminescence of PAAm-SiCQDs after processing. These results provide a plausible explanation for the fluorescence attenuation observed in PAAm-SiCQDs (Figure S17a).

**Figure S28**. TEM images showing the apparent size change of silicon carbide quantum dots before and after processing.

In a separate AuCl_3_-containing PAAm-SiCQDs composite, laser processing resulted in fluorescence enhancement rather than attenuation, as shown in Figure S17b. This enhancement is likely associated with laser-induced reduction of AuCl_3_ into Au-containing nanoparticles, which may contribute to metal-enhanced fluorescence of SiCQDs. In summary, composition-dependent fluorescence attenuation or enhancement was achieved in different PAAm-based hydrogel composites after femtosecond laser processing.
